# PWO proteins are associated with PRC2 since their emergence in vascular plants

**DOI:** 10.1111/nph.71172

**Published:** 2026-04-20

**Authors:** Ahamed Khan, Saqlain Haider, Abdoallah Sharaf, Alžbeta Kusová, Jan Skalák, Claire Jourdain, Martin Rennie, Petra Procházková Schrumpfová, Jan Hejátko, Daniel Schubert, Sara Farrona, Iva Mozgová

**Affiliations:** ^1^ Biology Centre, Institute of Plant Molecular Biology (IPMB) Czech Academy of Sciences 37005 České Budějovice Czech Republic; ^2^ Institute of Biology Freie Universität Berlin 14195 Berlin Germany; ^3^ School of Biological and Chemical Sciences, College of Science and Engineering University of Galway H91 TK33 Galway Ireland; ^4^ Centre for Chromosome Biology, Institute for Health Discovery and Innovation University of Galway H91 TK33 Galway Ireland; ^5^ Faculty of Science, National Centre for Biomolecular Research Masaryk University 61137 Brno Czech Republic; ^6^ Mendel Centre for Plant Genomics and Proteomics, Central European Institute of Technology (CEITEC), Masaryk University 62500 Brno Czech Republic; ^7^ School of Molecular Biosciences University of Glasgow G128QQ Glasgow UK

**Keywords:** Arabidopsis, CURLY LEAF (CLF), plant, Polycomb Repressive Complex 2 (PRC2), PWO, PWWP, *Selaginella*

## Abstract

- The Polycomb repressive complex 2 (PRC2), a conserved histone methyltransferase complex, plays a central role in transcriptional silencing across eukaryotes. Here, we investigate the evolution of PWWP‐DOMAIN INTERACTORS OF POLYCOMBS (PWOs), which interact with PRC2, and examine the conservation of the PWOs‐PRC2 interaction across plant evolution by comparing orthologs from the lycophyte *Selaginella moellendorffii* (Sm) and the flowering plant *Arabidopsis thaliana* (Arabidopsis; At).
- Phylogenetic data traced the presence of PWO proteins across plant lineages, while protein–protein interaction assays and AlphaFold predictions assessed PWO‐PRC2 interactions and structural conservation. Functional complementation assays confirmed PWOs' conserved functions.
- PWO proteins are present in vascular plants but absent in bryophytes and green algae. The ancestral clade of PWO proteins includes the spikemoss *Selaginella moellendorffii* (Sm) PWO orthologs SmPWOa and SmPWOb. PWO proteins from vascular plants can interact with PRC2 components from multiple species, including bryophytes, which naturally lack PWOs. The PWO‐PRC2 interaction is mediated by a conserved short C‐motif. Functional and molecular assays further demonstrate that SmPWOs retain their conserved functions in Arabidopsis.
- Our data suggest an evolutionarily conserved role for PWOs in modulating PRC2 activity and emphasize how lineage‐specific proteins associated with conserved chromatin‐modifying complexes may shape epigenetic control mechanisms during evolution.

## Introduction

The PWWP (Pro‐Trp‐Trp‐Pro) domain, a member of the Royal family of domains (Tudor, Chromo (chromatin‐binding), MBT (malignant brain tumor), and PWWP domains), functions as a recognition domain that binds both DNA and methylated lysine residues within histones (Qin & Min, 2014; Rona *et al*., 2016). PWWP domain‐containing proteins are usually take part in complexes associated with chromatin structure, facilitating crosstalk between different epigenetic marks, and thereby regulating gene expression (Qin & Min, 2014; Tan *et al*., 2018). Proteins carrying the PWWP domain are found in both plants and animals, where the PWWP domain occurs either as a single conserved domain or in a conserved combinatorial arrangement with other domains (Alvarez‐Venegas & Avramova, 2012). Among PWWP domain‐containing proteins, the PWWP‐DOMAIN INTERACTOR OF POLYCOMBS (PWO/PWWP) proteins are characterized by a distinct single N‐terminal PWWP domain without any additional structured domains (Hohenstatt *et al*., 2018; Tan *et al*., 2018).

In the flowering dicot model *Arabidopsis thaliana* (Arabidopsis, At), three PWO orthologs – AtPWO1, AtPWO2, and AtPWO3 – are present and play important roles in plant development. Homozygous *pwo* triple mutant plants (*pwo1;pwo2;pwo3*) are lethal at an early seedling stage, but milder phenotype defects are observed in *pwo* single and double mutant plants (Hohenstatt *et al*., 2018). PWO1 interacts with the evolutionarily conserved histone methyltransferase subunits of the Polycomb Repressive Complex 2 (PRC2), namely CURLY LEAF (CLF) and SWINGER (SWN) (Hohenstatt *et al*., 2018). These subunits contain a Su(var)3–9, Enhancer‐of‐zeste (E(z)), and Trithorax (SET) domain that catalyzes the deposition of H3K27me3 at specific loci, resulting in transcriptional gene silencing (Baile *et al*., 2022). A genetic interaction between *PWO1* and *CLF* exists – *pwo1;clf* enhances the *clf* phenotype, exacerbating upward leaf curling and affecting the transcription of PRC2 target genes (Hohenstatt *et al*., 2018). Recently, PWO1 was shown to bind the boundary regions of H3K27me3‐enriched compartment domains (CDs), which are topologically associated chromatin domains positioned at the nuclear periphery. In this context, PWO1 maintains the repressive structure of H3K27me3‐CDs (Yang *et al*., 2024). PWO1 also associates with the lamin‐like protein CROWDED NUCLEI 1 (CRWN1), controlling nuclear morphology and regulating partially overlapping sets of target genes in Arabidopsis (Mikulski *et al*., 2019). PWOs could therefore act as a bridge connecting H3K27me3‐CDs and CRWN1 at the nuclear periphery and contributing to a repressive chromatin state (Yang *et al*., 2024). In addition, PWOs have been identified as core components of the PEAT complex, which includes PWOs, AT‐rich interaction domain‐containing proteins (ARIDs), ENHANCER OF POLYCOMB‐RELATED proteins, and TELOMERE REPEAT BINDING proteins (TRBs). Although it was initially proposed that the PEAT complex maintains heterochromatin silencing through its interaction with histone deacetylases (Tan *et al*., 2018), more recently it has been shown to interact with histone acetyltransferases of the MYST family and UBIQUITIN PROTEASE 5 (UBP5) to induce gene expression by histone 4 (H4) acetylation and H2A deubiquitination (Zheng *et al*., 2023; Godwin *et al*., 2024). Despite PWO1's ability to contribute to different chromatin protein networks, the molecular mechanisms underlying the contrasting effects of the putative PWO‐associated protein complexes on chromatin structure remain elusive.

Emerging evidence shows that PRC2 is evolutionarily conserved in unicellular and multicellular eukaryotes (Schubert, 2019; Déléris *et al*., 2021; Sharaf *et al*., 2022; de Potter *et al*., 2023; Hisanaga *et al*., 2023). The PRC2 catalytic subunits CLF and SWN arose from an early gene duplication during land plant evolution. CLF represents the most ancestral paralog and is broadly conserved across land plants, while SWN emerged in angiosperms, with CLF and SWN paralogs present in both monocots and dicots (Shu *et al*., 2020; Vijayanathan *et al*., 2022). PRC2‐mediated gene silencing is crucial for cell differentiation, developmental transitions, and the establishment and maintenance of cell and organ identity in both plants and animals (Hennig & Derkacheva, 2009; de Lucas *et al*., 2016; Schuettengruber *et al*., 2017). PRC2 double mutants, such as *clf‐28 swn‐7*, exhibit severe developmental defects in Arabidopsis, characterized by the formation of masses of aerial tissues resembling cotyledonary or callus‐like structures (Chanvivattana *et al*., 2004; Mozgová *et al*., 2017; Shu *et al*., 2020). Loss of PRC2 also leads to severe developmental defects in basal land plants. In the moss *Physcomitrium patens*, loss of CLF results in apogamy, where a sporophyte‐like body emerges from a gametophytic vegetative cell (Okano *et al*., 2009). In addition to developmental genes, PRC2 also targets genes involved in environmental sensing and stress response, extending its functions beyond developmental control (Folsom *et al*., 2014; Vyse *et al*., 2022; Faivre *et al*., 2024; Zarif *et al*., 2024). A shift in PRC2 targeting from transposable elements (TEs) to protein‐coding genes has been proposed as the evolutionary innovation of PRC2 function in land plants (Hisanaga *et al*., 2023). Considering the significant innovations associated with the evolution of land plants (Donoghue *et al*., 2021), it is intriguing to ask how the functions of PRC2 have evolved to meet the demands for developmental and environmental acclimation in land plants. This raises important questions regarding the molecular adaptations and regulatory mechanisms that allowed PRC2 to incorporate additional accessory proteins, which may contribute to transcriptional regulation in terrestrial plants. PWOs, as components of both repressive (e.g. PRC2 complex) and activating (e.g. PEAT complex) regulatory complexes (Hohenstatt *et al*., 2018; Tan *et al*., 2018; Zheng *et al*., 2023; Godwin *et al*., 2024), may play key roles in transcriptional switches associated with environmental and developmental responses, adjusted during land plant evolution.

In this study, we address the evolution and function of PWO proteins, with a focus on the conservation of PWO‐PRC2 interaction. We show that PWOs emerge in lycophytes but are absent in bryophytes and green algae. To understand molecular interactions and functions of PWOs in plant evolution, we study *Selaginella moellendorffii* (Sm) SmPWOa and SmPWOb as representatives of early‐emerging PWOs. We demonstrate that, similar to AtPWO1, SmPWOa physically interacts with CLF, tethering CLF orthologs from different species to the subnuclear compartment where it localizes. In addition to the N‐terminal PWWP domain, we identify a novel conserved C‐motif that is required for PWO interaction with PRC2 catalytic subunits throughout evolution. Finally, we demonstrate that SmPWOs partially complement the *pwo1;pwo2* double mutant phenotype in Arabidopsis. Altogether, our data indicate that PWOs have been closely associated with PRC2 since their emergence in lycophytes, suggesting a deep evolutionary origin of their function in coordination with the Polycomb Group (PcG) repressive pathway.

## Materials and Methods

### Plant materials and growth conditions

All *Arabidopsis thaliana* (L.) Heynh. transgenic lines used in the current study were in Columbia (Col‐0) ecotype. *pwo1‐1* (Sail_342_C09; At3g03140) single mutant was crossed with *pwo2‐2* single mutant (Salk 136 093; At1g51745) to obtain the *pwo1‐1;pwo2‐2* double mutant (Hohenstatt *et al*., 2018). The homozygous mutants were confirmed by PCR‐based genotyping using primers P1–P8 (Supporting Information Table S1). Subsequently, the *Agrobacterium tumefaciens* (GV3101) harboring the pMDC85‐*2X35S*_*pro*_ *::SmPWOa‐GFP* and pMDC85‐*2X35S*_*pro*_ *::SmPWOb‐GFP* constructs (Curtis & Grossniklaus, 2003) were transformed into *pwo1‐1;pwo2‐2* double mutants using the floral dip transformation method (Karimi *et al*., 2007). The Col‐0, *pwo1‐1;pwo2‐2* double mutant, *2X35S*_*pro*_ *::SmPWOa‐GFP; pwo1‐1;pwo2‐2* and *2X35S*_*pro*_ *::SmPWOb‐GFP*; *pwo1‐1;pwo2‐2* were grown in pots containing peat, vermiculite, and perlite in a ratio of 5 : 1 : 1. The plants were grown under long day (LD) (16 h : 8 h, light : dark) conditions at 20°C : 18°C temperature regime under fluorescent lamps at 120 μmol m^−2^ s^−1^. At least 10 plants per genotype were used for flowering time analyses. Flowering time was scored by counting the number of days before the first flower opened. *In vitro* studies, such as root measurement, were carried out on 10‐d‐old seedlings grown on ½ Murashige & Skoog (MS) medium supplemented with 0.8% agar and 0.5% sucrose. Seeds were sterilized in 70% ethanol for 7 min and centrifuged, followed by sterilization in 100% ethanol for 5 min and subsequent centrifugation. The seeds were kept in a sterile hood to allow the residual ethanol to evaporate. Seeds were first stratified at 4°C for 3 d for breaking dormancy and synchronizing germination. Three‐week‐old soil‐grown *Nicotiana benthamiana* (Domin) H. Wheeler plants were used for agroinfiltration for transient gene expression.

### Homolog identification and phylogenetic analyses

The complete predicted proteome sequences of study organisms (Table S2) were obtained from the NCBI GeneBank (https://ncbi.nlm.nih.gov), UniProt‐Proteomes database (https://www.uniprot.org), and JGI (http://genome.jgi.doe.gov). The 1000 Plants project (OneKP) database (http://www.onekp.com) was an additional source for predicted proteome sequences inferred from transcriptomic data. Transcriptomic datasets were included only if they exhibited a BUSCO completeness score above 50%, ensuring sufficient coverage and reliability. The Arabidopsis PWO1 (*At3g03140*, Q9M9N3) reference amino acid sequence was used to search for all predicted proteomes using the Hidden Markov model (HMM)‐based tool jackhammer (Johnson *et al*., 2010). Evolutionary genealogy of genes: non‐supervised Orthologous Groups (eggNOG) mapper was used for hierarchical resolution of orthology assignments (Huerta‐Cepas *et al*., 2019). Only eggNOG‐hits with ENOG5028MA3 and ENOG5028N12 were selected. Finally, the SMART and Pfam databases were employed to identify conserved domains present in PWOs from different organisms (Letunic & Bork, 2018; El‐Gebali *et al*., 2019), both SMART and Pfam databases were merged, redundant domains were filtered out, and the Hidden Markov model (HMM)‐based tool hmmscan (https://github.com/EddyRivasLab/hmmer) was used to scan domain architecture.

All identified ortholog sequences were aligned using Mafft software (Katoh & Standley, 2013), and ambiguously aligned regions were excluded for further analysis using trimAl software (Capella‐Gutiérrez *et al*., 2009). Alignments were tested using ProtTest v3 (Darriba *et al*., 2010) to choose an appropriate model for nucleotide substitution. Two separate Maximum likelihood (ML) phylogenetic trees were computed using RAxML‐NG (Kozlov *et al*., 2019) and Iq‐Tree 2 (Minh *et al*., 2020) software. ML analyses were performed using 1000 bootstrap replicates. The supporting values from both software were noted on the ML‐rooted tree. The phylogenetic tree was rooted with Lycopodiopsida sequences, which were considered as the emerging point of the PWO family.

### Cloning and plasmid preparation

The full‐length coding sequences of SmPWOa (P9‐P10), SmPWOb (P11‐P12), SmCLF (P13‐P14), and PpCLF (P15‐P16) were amplified (Table S1) from a total cDNA library prepared from *Selaginella moellendorffii* Hieron. and *Physcomitrium patens* (Hedw.) Mitt. They were then cloned into pJET1.2/blunt (CloneJET™ PCR Cloning Kit; Thermo Scientific #K1231, #K1232; Waltham, MA, USA). Subsequently, all pJET1.2 clones were validated by colony PCR, plasmid digestion, and Sanger sequencing, and used as templates for further cloning. Similarly, *SmPWOa* (P17‐P18), *SmPWOb* (P19‐P20), *SmCLF* (P21‐P22), and *PpCLF* (P23‐P24) cDNAs were cloned into pDONR221 (Table S1), followed by subcloning into destination vectors using the Gateway™ technology (Invitrogen; Carlsbad, CA, USA) (pGBKT7‐GW, pGADT7‐GW, pMDC7:i35S‐GFP, pMDC7:i35S‐mCherry, pMDC85‐GFP) (Bleckmann *et al*., 2009; Lu *et al*., 2009). Additionally, the *ARR1* (*AT3G16857*) and *ARR2* (*AT4G16110*) cDNAs were amplified using primers P25‐P26 (*ARR1*) and P27‐P28 (*ARR2*). The resulting fragments were recombined into the donor vector pDONR™/Zeo, following the manufacturer's protocol for the Gateway cloning system (Thermo Fisher Scientific), and subsequently subcloned into the pB7WGR2 destination vector (Karimi *et al*., 2007) (Table S1). In‐Fusion Snap Assembly (Takara; Kyoto, Japan) was used for the *SmPWOa* (P29‐P30) and *SmPWOb* (P31‐P32) cDNAs cloning into the pMDC85 vector (Curtis & Grossniklaus, 2003) (Table S1). Arabidopsis gene clones, including *pMDC7:i35S‐AtPWO1‐GFP*, *pMDC7:i35S‐AtPWO1‐mCherry*, *pGBKT7‐AtCLFΔSET*, and *pGBKT7‐AtSWNΔSET*, were generated before (Hohenstatt *et al*., 2018; Mikulski *et al*., 2019). For C‐motif deletion, mutated primers were used to amplify *AtPWO1* (P33‐P34) and *SmPWOa* (P35‐P36) cDNAs without the C‐motif, followed by cloning into pDONR221 vectors and subsequent subcloning into pGBKT7‐GW, pGADT7‐GW, pMDC7:i35S‐GFP, and pMDC7:i35S‐mCherry.

### Yeast two‐hybrid assays

For yeast two‐hybrid (Y2H) assays, the *S. cerevisiae* strain AH109 was used to co‐transform both pGADT7‐Gal4‐AD and pGBKT7‐Gal4‐BD constructs in various combinations, following the protocol outlined in the Yeast Protocols Handbook (version no. 325, PR973283 21; Clontech; San Jose, CA, USA). Transformed yeast cells were selected on synthetically defined (SD) medium lacking leucine (L) and tryptophan (T), supplemented with adenine (A) and histidine (H); (SD/‐Trp/‐Leu/+Ade/+His) at 30°C for 2–3 d. Interaction assays were conducted on SD medium with both low stringency (SD/‐Trp/‐Leu/+Ade/‐His) and high stringency selection plates (SD/‐Trp/‐Leu/‐Ade/‐His). Co‐transformation involving empty Gal4‐AD and Gal4‐BD constructs, as well as empty bait with prey constructs, served as controls.

### Subcellular localization and FRET assays

*Agrobacterium tumefaciens* (GV3101) was transformed with pMDC7 (estradiol‐inducible) and pMDC85 clones tagged with GFP/mCherry employing the Freeze–Thaw method (Weigel & Glazebrook, 2006). After transformation, the colonies were allowed to grow for 2 d on yeast extract broth (YEB) (Y1625; Sigma‐Aldrich) medium plates. Transformed colonies were then cultured in YEB medium overnight, followed by subculture for an additional 3–4 h. Subsequently, the cultures were harvested and resuspended in infiltration medium (containing 10 mM MgCl_2_ (M8266; Sigma‐Aldrich), 10 mM MES (M3671; Sigma‐Aldrich) [pH 5.6], and 200 μM acetosyringone (D134406; Sigma‐Aldrich)) to an optical density (OD) of 0.6 and left at room temperature for 1 h. Bacteria were then infiltrated into 3‐ to 4‐wk‐old *N. benthamiana* leaves using 2‐ml syringes and left for 48 h. The induction buffer (Beta‐estradiol (E2758; Sigma‐Aldrich) solution in 0.1% (v/v) TWEEN‐20 (P1379; Sigma‐Aldrich)) was sprayed on the abaxial side of the leaves. At 6–8 h post‐induction, infiltrated leaves were subjected to confocal microscopy (Olympus FV3000). Image analysis was carried out using Fiji/ImageJ software (Schindelin *et al*., 2012) (National Institutes of Health).

For FLIM‐FRET experiments, all plasmids were transiently expressed in *Nicotiana tabacum* L. (SR1 Petit Havana) leaf epidermal cells using the infiltration procedures described by Voinnet *et al*. (2000). Confocal microscopy was performed using a laser scanning confocal imaging microscope Zeiss LSM 780 AxioObserver equipped with an external In Tune laser (488–640 nm, < 3 nm width, pulsed at 40 MHz, 1.5 mW) and a C‐Apochromat ×63 water objective with NA 1.2. FLIM‐FRET data acquisition was conducted using an HPM‐100‐40 Hybrid Detector from Becker and Hickl GmbH, which includes Simple‐Tau 150N (Compact TCSPC system based on SPC‐150N) with a DCC‐100 detector controller for photon counting. Zen 2.3 light version from Zeiss was utilized for processing confocal images. The acquisition and analysis of FLIM data involved the utilization of Spcm 64 v.9.8 and SPCImage v.7.3 from Becker and Hickl GmbH, respectively. A multiexponential decay model was employed for fitting the data. For the prediction of NLSs in PWO proteins, DeepLoc‐2.0 (Thumuluri *et al*., 2022) was utilized. This tool is designed to predict the subcellular localization of proteins based on their amino acid sequences. Typically, threshold values closer to 1 indicate higher confidence in the prediction, while values closer to 0 indicate lower confidence.

### Alphafold2 (AF2) analyses

Protein structures and interaction interface were predicted using Ucsf ChimeraX (Pettersen *et al*., 2021; Meng *et al*., 2023), utilizing its built‐in tool for structure prediction via the AF2 server (Evans *et al*., 2021; Jumper *et al*., 2021). Each AF2 predicted file produced results with pdb and json files, graph for sequence homology coverage, pLDDT plot and 5 rank model PEA plots. The PDB files contain the structures predicted by ColabFold, while the .json file contains the individual scores for each amino acid in the predicted structure. Moreover, PAE plots show the uncertainty in the distance prediction between individual protein domains represented with a color code ranging from blue (0–15 Å) to red (15–30 Å). For the analysis, the pLDDT, PAE and ipTM have been taken into consideration and results were visualized using ChimeraX (Pettersen *et al*., 2021; Meng *et al*., 2023).

### Gene expression analyses

For expression analyses, total RNA was extracted from 10‐d‐old seedlings grown on ½MS media at ZT16 using the Thermo Scientific kit according to the manufacturer's instructions. Two micrograms of total RNA were used to synthesize first‐strand cDNA using the RevertAid First strand cDNA synthesis kit by Thermo Scientific. The PCR amplification from cDNA was carried out using Takyon™ No ROX SYBR 2X MasterMix blue dTTP (Eurogentec; Liège, Belgium) using the Primers P37‐P44 (Table S1). *TIP41* (*TAP42 INTERACTING PROTEIN OF 41 kDa*) (P45‐P46) was used as an internal control (Table S1). Error bars show the SD of three biological replicates.

### Chromatin immunoprecipitation assay

Chromatin immunoprecipitation (ChIP) was performed using 2 g of seedlings from each genotype as previously described (Godwin *et al*., 2024). Briefly, 10‐d‐old seedlings were harvested and cross‐linked using 1% formaldehyde under vacuum (Fisher vacuum pump). Chromatin was then extracted and fragmented to an average size of 200–500 bp using a Bioruptor® Pico (Diagenode, Liège, Belgium). Protein concentration was quantified using the Pierce™ BCA Protein Assay Kit, and 100 μg of protein was used per sample. For pre‐clearing, 30 μl of Protein A Dynabeads per sample were used before immunoprecipitation (IP). IP was carried out using 60 μl of Protein A Dynabeads and 5 μl of antibody per sample in ChIP dilution buffer, followed by incubation at 4°C overnight. The antibody used for ChIP‐qPCR was anti‐H3K27me3 (ab6002; Abcam; Cambridge, UK). Following IP, chromatin complexes were sequentially washed with low‐salt, high‐salt, LiCl, and TE buffers. Chromatin was then eluted and reverse cross‐linked with 5 M NaCl at 65°C overnight. DNA was recovered by phenol : chloroform:isoamyl alcohol extraction (25 : 24 : 1, pH 8.05), followed by ethanol precipitation. Input DNA was diluted 1 : 10, and 2 μl of immunoprecipitated DNA was used for quantitative PCR (qPCR).

ChIP‐qPCR was performed using a LightCycler® 480 Instrument (Roche, Basel, Switzerland) with Takyon™ No ROX SYBR 2× MasterMix Blue dTTP (Eurogentec). Primers used to amplify the regions are mentioned in the Table S1 (P47‐P61). Two biological replicates and three technical replicates were used for each experiment. Statistical significance was assessed using a Student's *t*‐test. Error bars represent ± SD (SD). Significance levels are indicated as ***, *P* ≤ 0.001; **, *P* ≤ 0.01; *, *P* ≤ 0.05. The percentage of input was calculated as previously described (Godwin *et al*., 2024), and *AT5G13440* was used as a negative control.

### Nuclear morphology analyses

Nuclei isolation was conducted using 10‐d‐old seedlings of Col‐0 (WT), *pwo1‐1;pwo2‐2* double mutants, *2X35S*_*pro*_ *::SmPWOa‐GFP; pwo1‐1;pwo2‐2* and *2X35S*_*pro*_ *::SmPWOb‐GFP*; *pwo1‐1;pwo2‐2* genotypes following the protocol described by Kalyanikrishna *et al*., 2020 (Kalyanikrishna *et al*., 2020). Fluorescence imaging of the isolated nuclei stained with DAPI was performed using a Leica SP8 confocal microscope, with a 405 nm diode laser for DAPI excitation. Z‐stacks of 40–60 images were acquired with a step size of 0.25 μm, maintaining consistent imaging settings across genotypes. Nuclear area and circularity index were measured using Fiji/ImageJ (Schindelin *et al*., 2012), with z‐stacks analyzed according to Kalyanikrishna *et al*., 2020 (Kalyanikrishna *et al*., 2020). More than 100 nuclei per genotype were analyzed, and for each genotype, three technical replicates and two biological replicates were used to conduct the experiment.

## Results

### PWO proteins are found in vascular plants and form four major clades

**Fig. 1:**
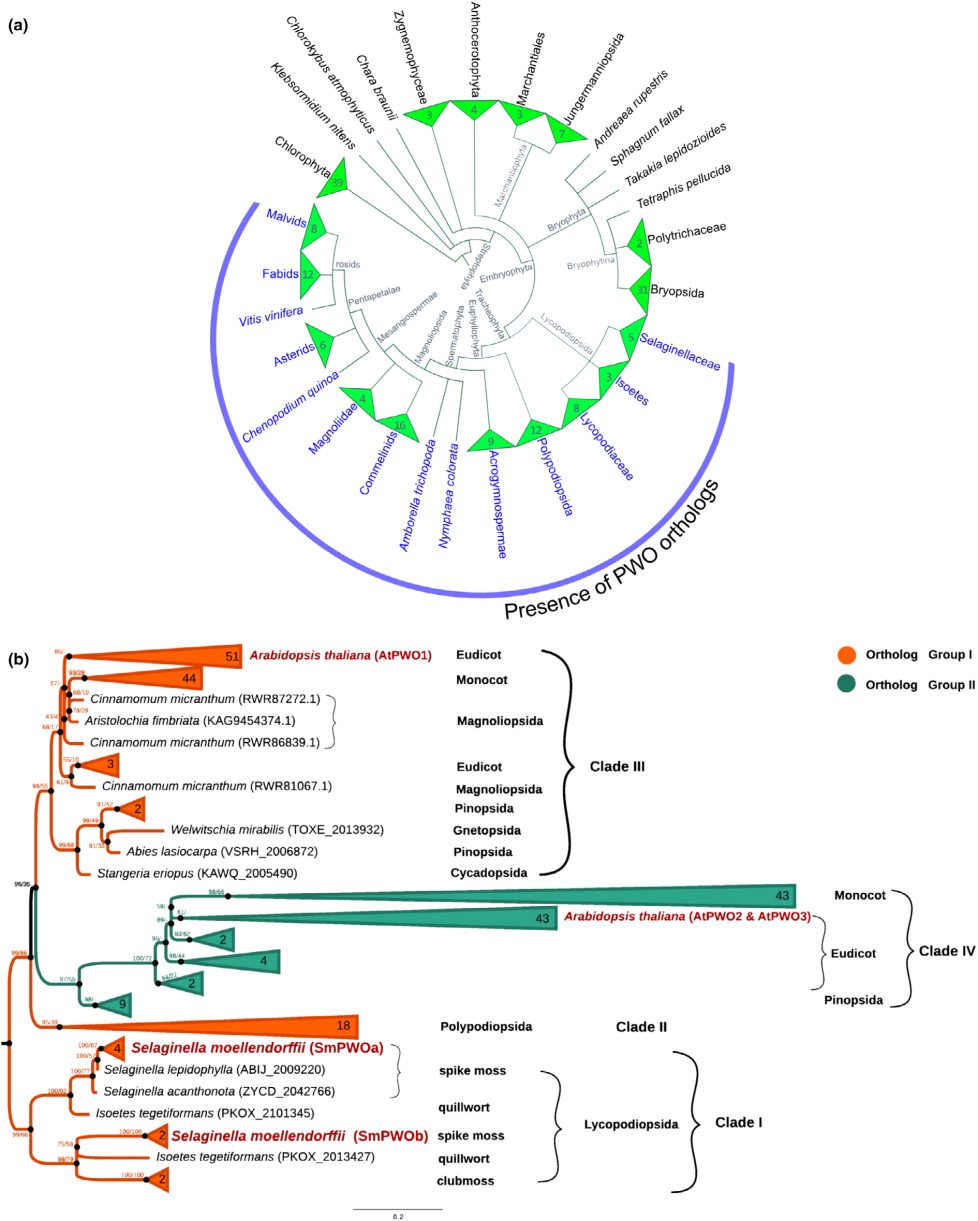
The evolution and distribution of PWO proteins in Viridiplantae. (a) Cladogram showing the diversity of PWO protein orthologs across the green lineage. Blue line indicates the presence of PWO orthologs. The numbers in collapsed clade triangles indicate the numbers of PWO proteins including isoforms identified within each taxonomic group (Supporting Information Table S3). (b) Maximum likelihood (ML) phylogenetic tree of PWO protein orthologs showing separation into four clades (Clades I–IV). The tree was constructed using full‐length protein sequences. Numbers shown at internal nodes indicate branch support values expressed as percentages (IQ‐TREE/RAxML‐NG), whereas numbers in colored collapsed triangles indicate the number of PWO protein sequences contained within each collapsed clade. Orange and green branches represent ortholog Groups I and II, respectively. Roman numerals (I–IV) denote the four major phylogenetic clades. The tree was rooted using sequences of Lycopodiopsida PWO orthologs, and the Bar represents 0.2 amino acid substitutions per site.

To identify PWO orthologs in the green lineage and address their evolutionary relationships, we used a previously established bioinformatic pipeline (Sharaf *et al*., 2022). We searched for orthologs of AtPWO1 in 183 species representing Chlorophyta and Streptophyta, including algae and land plants. PWO orthologs were identified in 66 species (36%) (Table S2), all representing land plants. Interestingly, PWO orthologs were absent in non‐vascular plant groups but were well conserved in vascular plants (Tracheophyta), first appearing in extant lycophytes (Lycopodiopsida) (Fig. 1a; Table S2).

The identified PWO sequences clustered into four major clades (Clades I–IV) (Fig. 1b; Table S3). Moreover, orthology assignment using the EggNOG database (Huerta‐Cepas *et al*., 2019) identified two orthology groups: ortholog group I (ENOG5028MA3) and ortholog group II (ENOG5028N12) (Fig. 1b). The orthologs of the first group clustered into three clades (Fig. 1b). Clade I (‘ancestral’ PWO clade) comprised lycophyte orthologs, where orthologs of clubmosses, spike mosses, and quillworts diverged into two subclades. One of the subclades included *S. moellendorffii* SmPWOa paralogs. The other subclade included the SmPWOb paralogs. PWO Clade II included Polypodiopsida (ferns and horsetails) orthologs, grouped into a monophyletic clade at the root of the remaining clades (Fig. 1b). PWO Clade III clustered at the crown of the tree and contained orthologs of seed plants – gymnosperms (Cycadopsida, Pinopsida, Gnetopsida) and angiosperms (Magnoliopsida, monocots, and eudicots). This clade included the queried AtPWO1. Finally, Clade IV, which belongs to the second orthology group, clustered at the root of Clade III and contained orthologs of seed plants (Pinopsida, monocots, and eudicots), including AtPWO2 and AtPWO3.

### PWOs structural prediction shows an evolutionarily conserved PWWP domain and a short C‐motif

**Fig. 2:**
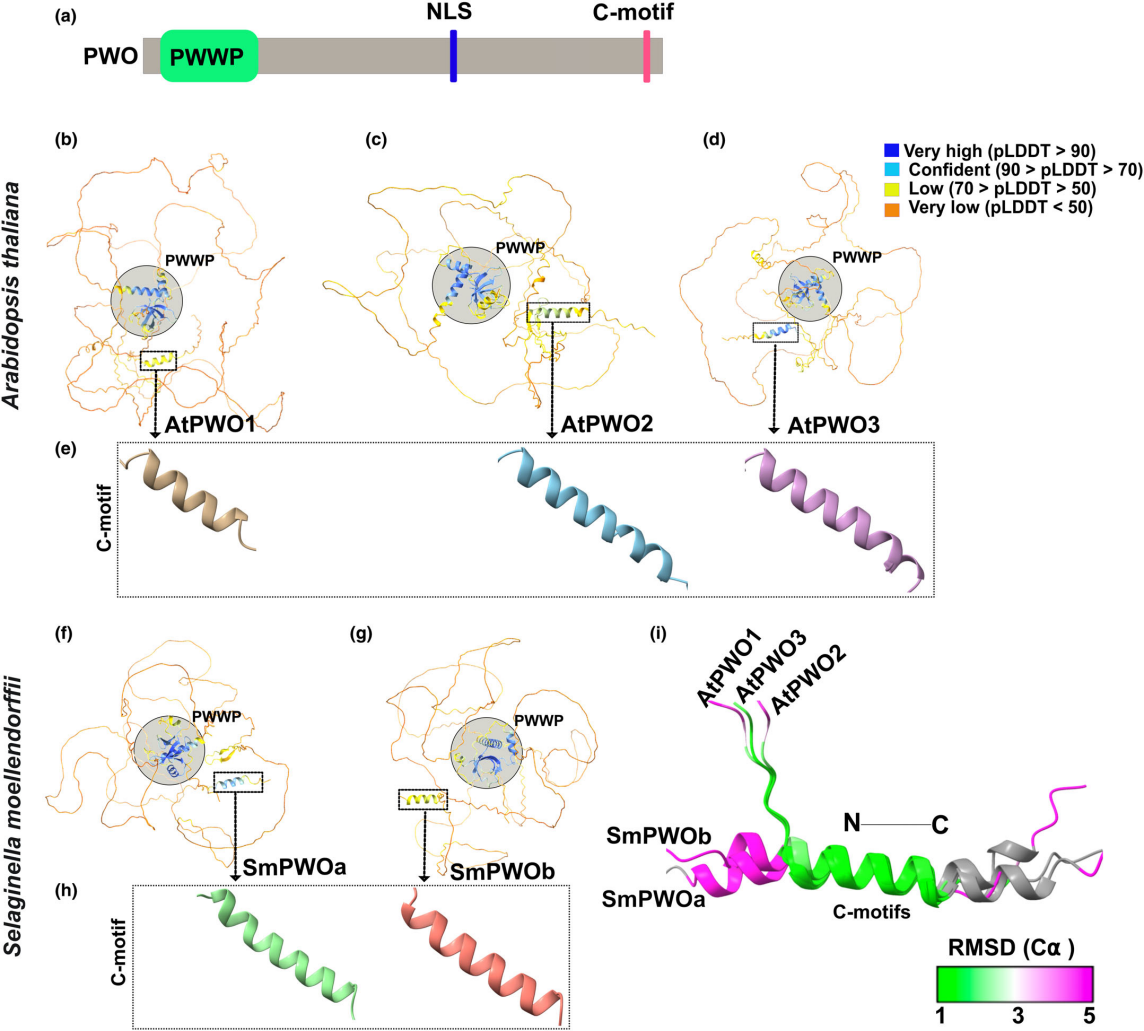
Alphafold2‐based protein structure and domain/motif prediction of PWO proteins. (a) Schematic representation of full‐length PWO proteins showing the conserved N‐terminal PWWP domain and C‐motif. PWOs have one or several predicted nuclear localization signal(s) (NLS), here represented by a single one. (b–e) Structure prediction of (b–d). Arabidopsis PWO orthologs (AtPWO1, AtPWO2, AtPWO3) including their (e). C‐motif (boxed). (f–h) *Selaginella moellendorffii* PWO orthologs (SmPWOa, SmPWOb), highlighting (f–g) their PWWP domain, and (h). C‐motif (boxed). The predicted full‐length PWO structures are depicted with per‐residue confidence scores (pLDDT) represented by different colors, ranging from 0 to 100, where regions with pLDDT below 50 may be considered unstructured. Different pseudocolors are used to depict the C‐motif (e, h). (i) Superposition of predicted C‐motifs from Arabidopsis and *S. moellendorffii* PWO orthologs on the AtPWO1 C‐motif (used as reference). The Root Mean Square Deviation of Alpha Carbon atoms (RMSD (C α)) color range is displayed, with green representing values of 1 Å or less indicating high structural similarity, white for intermediate values *c*. 3 Å, and magenta for an RMSD of 5 Å indicating the least structural similarity.

PWO proteins share (Fig. 2a) a conserved N‐terminal PWWP domain including a SWWP motif, a variation of this domain (Fig. S1) (Qin & Min, 2014; Rona *et al*., 2016). In addition, we identified one or more predicted nuclear localization signals (NLS) in the central region (Table S4) and a conserved sequence motif at the C‐terminus, referred to as the C‐motif from here on. The 3D structures of full‐length PWO sequences from two species, representing three clades of ortholog groups I–II, were predicted using Alphafold2 (AF2) (Jumper *et al*., 2021; Varadi *et al*., 2024). PWOs of the dicot Arabidopsis represented Clade III (AtPWO1) and Clade IV (AtPWO2, AtPWO3) (Fig. 2b–d; Table S5). PWOs of the spike moss *S. moellendorffii* (SmPWOa, SmPWOb; Table S5) represented the ancestral Clade I (Fig. 2f,g). The most structured regions of the PWOs (predicted Local Distance Difference Test (pLDDT) > 70) were within the N‐terminal PWWP domain (Figs 2b–d,f,g, S2A,B) and the short C‐motif, which folded into a short alpha‐helical structure (Fig. 2e,h). Outside of these regions, AtPWOs and SmPWOs exhibited long stretches of low confidence structure (per‐residue model confidence score (pLDDT) < 50), consistent with intrinsically disordered regions (IDRs) (Figs 2b–d,f,g, S2A) (Erdős & Dosztányi, 2024).

The predicted PWWP domains of PWO orthologs from Arabidopsis (Fig. S3A–E) and *S. moellendorffii* (Fig. S3A,B,F,G) corresponded to the canonical structure of PWWP domains (Wu *et al*., 2011; Qin & Min, 2014) with five antiparallel β‐strands (β1–β5) arranged in a β‐barrel, followed by three α‐helices. The position of α0 was variable, while α1 and α2 consistently formed after the β‐barrel structure (Fig. S3C–G). Superimposition of the PWWP domains of Arabidopsis and *S. moellendorffii* PWO orthologs showed structural conservation between AtPWO1 and AtPWO2‐3, as well as SmPWOa‐b (Fig. S3H; Table S6).

The predicted C‐motif from Arabidopsis and *S. moellendorffii* PWOs also exhibited significant structural similarity when superimposed onto the AtPWO1 C‐motif (Fig. 2I; Table S7). Moreover, prediction of 18 more PWO sequences representing different species from all four PWO clades confirmed the consistent presence of the C‐motif (Fig. S4A). Superposing all the selected C‐motifs onto the AtPWO1 C‐motif highlighted structural conservation (Fig. S4B; Table S7). Multiple sequence alignment showed a high level of amino acid (aa) conservation within the C‐motif, enriched with hydrophobic aa residues (Φ) (CΦPΦK/RΦΦΦXRΦXEXΦ) (Fig. S5).

### AtPWO1 and SmPWOs form nuclear speckles and colocalize in the same subnuclear space within plant nuclei

**Fig. 3:**
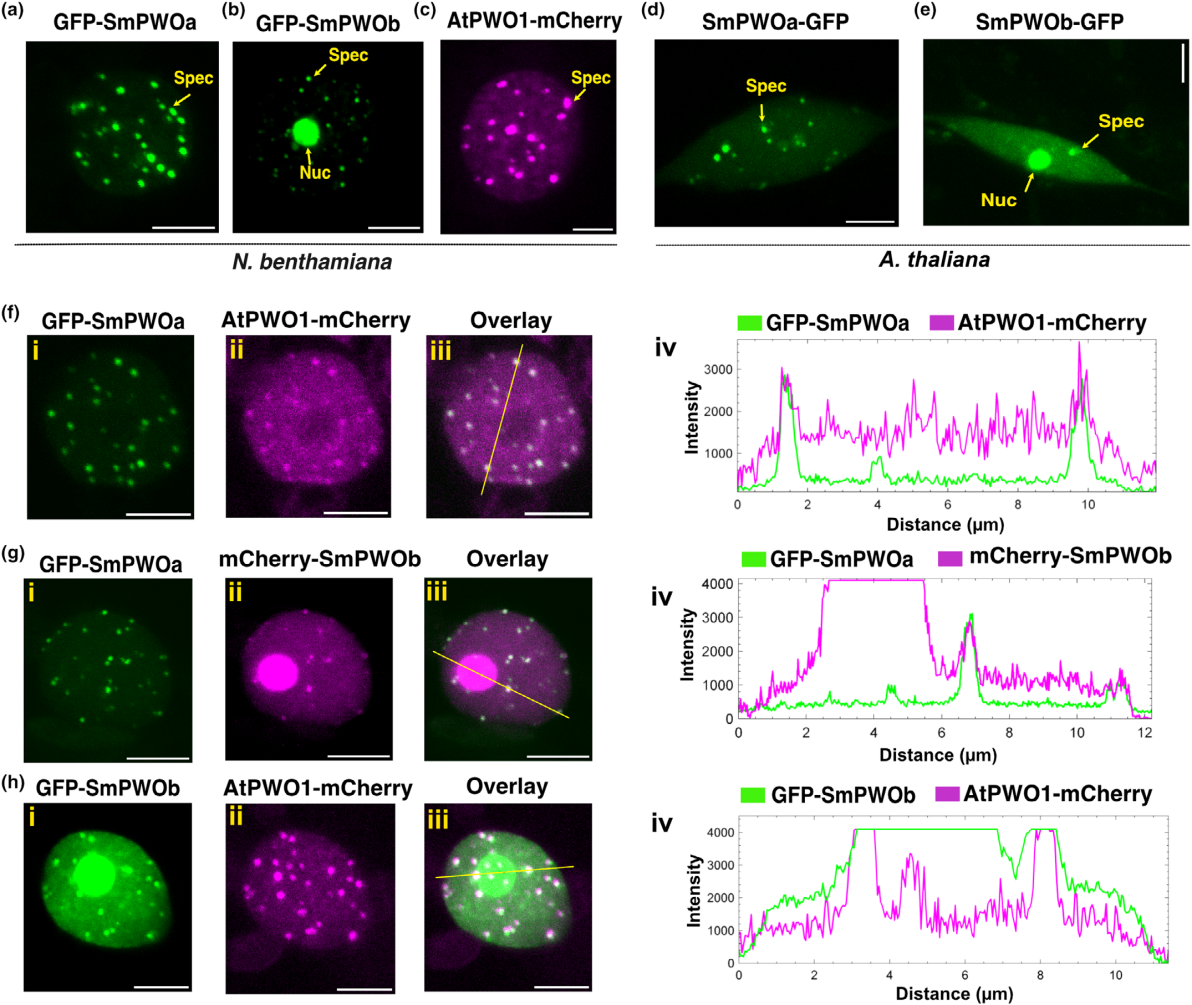
*In planta* subcellular localization of PWOs. (a–c) Representative confocal microscopy images of *Nicotiana benthamiana* leaf nuclei, infiltrated with: (a) *i35S*_*pro*_ *::GFP‐SmPWOa*, (b) *i35S*_*pro*_ *::GFP‐SmPWOb*, and (c) *i35S*_*pro*_ *::AtPWO1‐mCherry*. (d, e) Transgenic Arabidopsis lines expressing (d) *2X35*_*pro*_ *::SmPWOa‐GFP* and (e) *2X35*_*pro*_ *::SmPWOb‐GFP* in the *pwo1‐1;pwo2‐1* double mutant background in root tissues. The most prominent subnuclear structures are marked with arrows. ‘Spec’—speckles, and ‘Nuc’—nucleolus. (f–h) Representative confocal microscopy images of nuclei of *N. benthamiana* leaf co‐infiltrated with: (fi–iii) *i35S*_*pro*_ *::GFP‐SmPWOa* and *i35S*_*pro*_ *::AtPWO1‐mCherry*, (gi–iii) *i35S*_*pro*_ *::GFP‐SmPWOa* and *i35S*_*pro*_ *::mCherry‐SmPWOb*, (hi–iii) *i35S*_*pro*_ *::GFP‐SmPWOb* and *i35S*_*pro*_ *::AtPWO1‐mCherry*. (f–hiv). Profiles of mCherry and GFP fluorescence intensities along the yellow line indicated in (f–hiii). Bars, 5 μm.

The predicted structures of PWO proteins contained long stretches of IDRs (Figs 2b–d,f,g, S2), which are often associated with a high propensity to form condensates (Solis‐Miranda *et al*., 2023). Additionally, one or more NLSs (Table S4) located within the middle domain of the PWO proteins suggested their localization within the nucleus. Fluorescently tagged AtPWO1 and SmPWOs transiently expressed in *N. benthamiana* showed that GFP‐SmPWOa (Fig. 3a) and GFP‐SmPWOb (Fig. 3b), as well as AtPWO1‐mCherry (Fig. 3c; Hohenstatt *et al*., 2018), form speckles in the nucleoplasm. Notably, GFP‐SmPWOb, unlike GFP‐SmPWOa or AtPWO1‐GFP, localized to the nucleolus (Fig. 3a–c). The localization of stably expressed SmPWOa‐GFP and SmPWOb‐GFP in Arabidopsis (Fig. 3d,e) resembled that in *N. benthamiana* (Fig. 3a,b). The co‐expression of GFP‐SmPWOa with AtPWO1‐mCherry (Fig. 3f) or mCherry‐SmPWOb (Fig. 3g) resulted in the colocalization of the nucleoplasmic speckles. Similarly, the co‐expression of GFP‐SmPWOb and AtPWO1‐mCherry showed colocalization of speckles within the nucleoplasm (Fig. 3h). Additionally, SmPWOb also localized in the nucleolus, where it colocalized neither with SmPWOa nor AtPWO1 (Fig. 3g,h). Importantly, mCherry expressed alone did not form speckles and was excluded from the nucleolus, indicating that PWOs are needed to promote the speckle formation and/or nucleolar localization (Fig. S6A–C). Overall, SmPWOs and AtPWO1 form nucleoplasmic speckles that share the same subnuclear space, while SmPWOb additionally localizes to the nucleolus.

### PWOs tether CLF from the nucleoplasm to sub‐compartments in plant nuclei

**Fig. 4:**
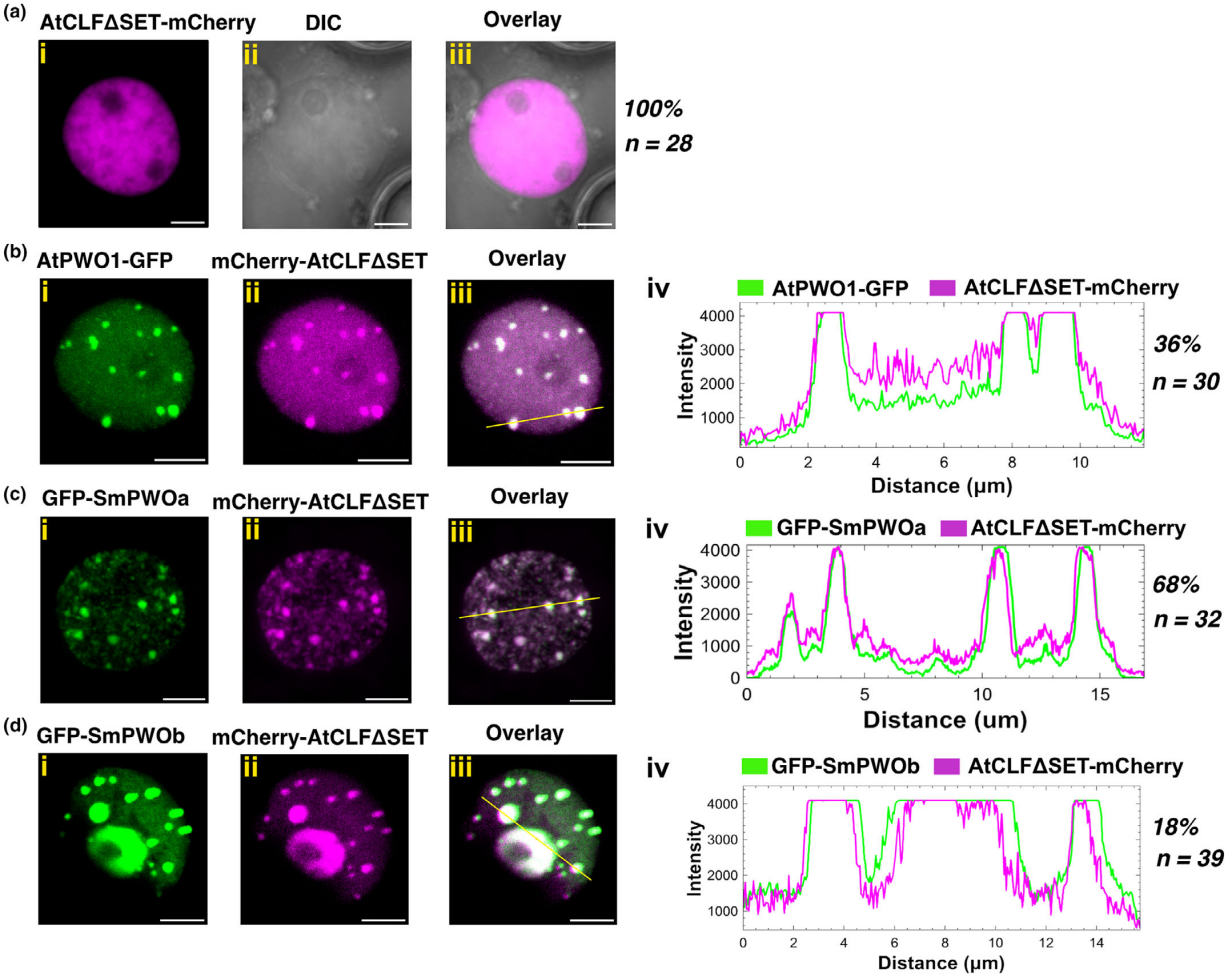
Colocalization of *Arabidopsis thaliana* and *Selaginella moellendorffii* PWOs with AtCLF. Representative confocal microscopy images showing nuclei of *Nicotiana benthamiana* leaf cells infiltrated with (ai–iii) *i35S*_*pro*_ *::mCherry‐AtCLFΔSET*, or co‐infiltrated with (bi–iii) *i35S*_*pro*_ *::AtPWO1‐GFP* and *i35S*_*pro*_ *::mCherry‐AtCLFΔSET*, (ci–iii) *i35S*_*pro*_ *::GFP‐SmPWOa* and *i35S*_*pro*_ *::mCherry‐AtCLFΔSET* and (di–iii) *i35S*_*pro*_ *::GFP‐SmPWOb* and *i35S*_*pro*_ *::mCherry‐AtCLFΔSET*. (b–div) Profiles of mCherry and GFP fluorescence intensities along the yellow line in (b–diii). Percentage of nuclei with the observed pattern vs total number of analyzed nuclei (n) is indicated on the right. ‘ΔSET’ denotes the deletion of SET domain. Bars, 5 μm.

Previously, AtPWO1 was shown to interact with CLF, the catalytic subunit of PRC2, and to tether AtCLF to nuclear speckles (Hohenstatt *et al*., 2018). This led us to investigate whether the tethering of CLF to PWOs‐derived nuclear speckles is conserved across PWOs and CLF orthologs in other species. When expressed alone in *N. benthamiana*, mCherry‐AtCLFΔSET, which has previously been shown to display identical localization and interacting behavior as the full‐length protein (Hohenstatt *et al*., 2018; Mikulski *et al*., 2019; Godwin *et al*., 2024), resulted in diffuse nuclear localization, but distinct nuclear speckles were never observed (Fig. 4a). By contrast, co‐expressing mCherry‐AtCLFΔSET with AtPWO1‐GFP changed the nuclear distribution of mCherry‐AtCLFΔSET in 36% of the nuclei, in which mCherry‐AtCLFΔSET colocalized with AtPWO1‐GFP speckles (Figs 4b, S7A). The co‐expression of GFP‐SmPWOa and mCherry‐AtCLFΔSET resulted in the formation of mCherry‐AtCLFΔSET speckles that overlapped with SmPWOa speckles in 68% of nuclei (Figs 4c, S7B). Similarly, co‐expression of GFP‐SmPWOb and mCherry‐AtCLFΔSET resulted in the formation of mCherry‐AtCLFΔSET speckles, although only in 18% of the nuclei (Figs 4d, S7C–E) and, interestingly, promoted the localization of mCherry‐AtCLFΔSET to the nucleolus in 90% of the nuclei (Figs 4d, S7C,D). Thus, the presence of PWOs potentiates the relocation of AtCLF within the nucleus.

**Fig. 5:**
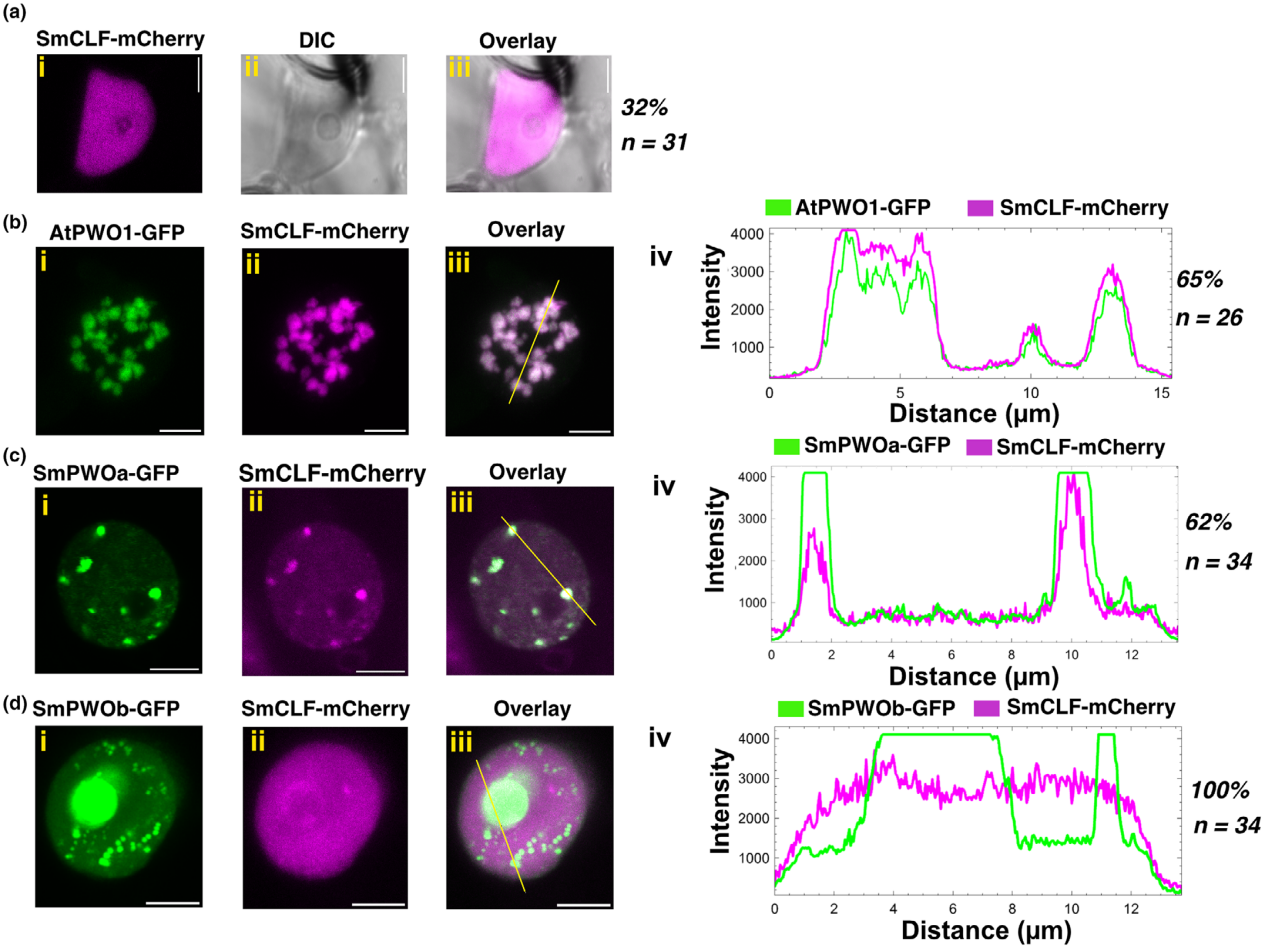
Colocalization of *Selaginella moellendorffii* (Sm) CLF with AtPWO1, SmPWOa, and SmPWOb in *Nicotiana benthamiana*. Representative confocal microscopy images showing nuclei of *N. benthamiana* leaf cells infiltrated with (ai–iii) *i35S*_*pro*_ *::SmCLF‐mCherry*, and co‐infiltrated with (bi–iii) *i35S*_*pro*_ *::AtPWO1‐GFP* and *i35S*_*pro*_ *::SmCLF‐mCherry*, (ci–iii) *i35S*_*pro*_ *::GFP‐SmPWOa* and *i35S*_*pro*_ *::SmCLF‐mCherry*, and (di–iii) *i35S*_*pro*_ *::GFP‐SmPWOb* and *i35S*_*pro*_ *::SmCLF‐mCherry*. (b–div) Profiles of mCherry and GFP fluorescence intensities along the yellow line in (b–diii). Percentage of nuclei with the observed pattern vs total number of analyzed nuclei (*n*) is indicated on the right. Bars, 5 μm.

Next, we asked whether AtPWO1 and SmPWOs could recruit SmCLF to PWO speckles. The localization of SmCLF‐mCherry alone showed uniform distribution within the nucleoplasm in *c*. 32% of nuclei (Fig. 5a), while the remaining nuclei showed strong accumulation of the SmCLF‐mCherry signal (Fig. S8A). Notably, this accumulation did not resemble the typical speckle pattern (Figs 5a, S8A). When AtPWO1‐GFP and SmCLF‐mCherry were co‐expressed, distinct patches of SmCLF‐mCherry overlapping with AtPWO1‐GFP were formed in 65% of nuclei (Fig. 5b). The remaining 35% of nuclei showed evenly distributed SmCLF‐mCherry, which was not concentrated into AtPWO1‐GFP speckles (Fig. S8B). Similarly, co‐expression of GFP‐SmPWOa and SmCLF‐mCherry resulted in 62% of nuclei displaying SmCLF‐mCherry speckles (Fig. 5c), while 23% of nuclei showed small patches of SmCLF‐mCherry with no substantial colocalization with GFP‐SmPWOa (Fig. S8C). The remaining 15% of nuclei showed a marginal overlap of GFP‐SmPWOa speckles with SmCLF‐mCherry (Fig. S8D). The co‐expression of GFP‐SmPWOb and SmCLF‐mCherry led to a subtle change in the localization of SmCLF‐mCherry from the nucleoplasm to the nucleolus, with no distinct formation of SmCLF‐mCherry speckles (Fig. 5d).

**Fig. 6:**
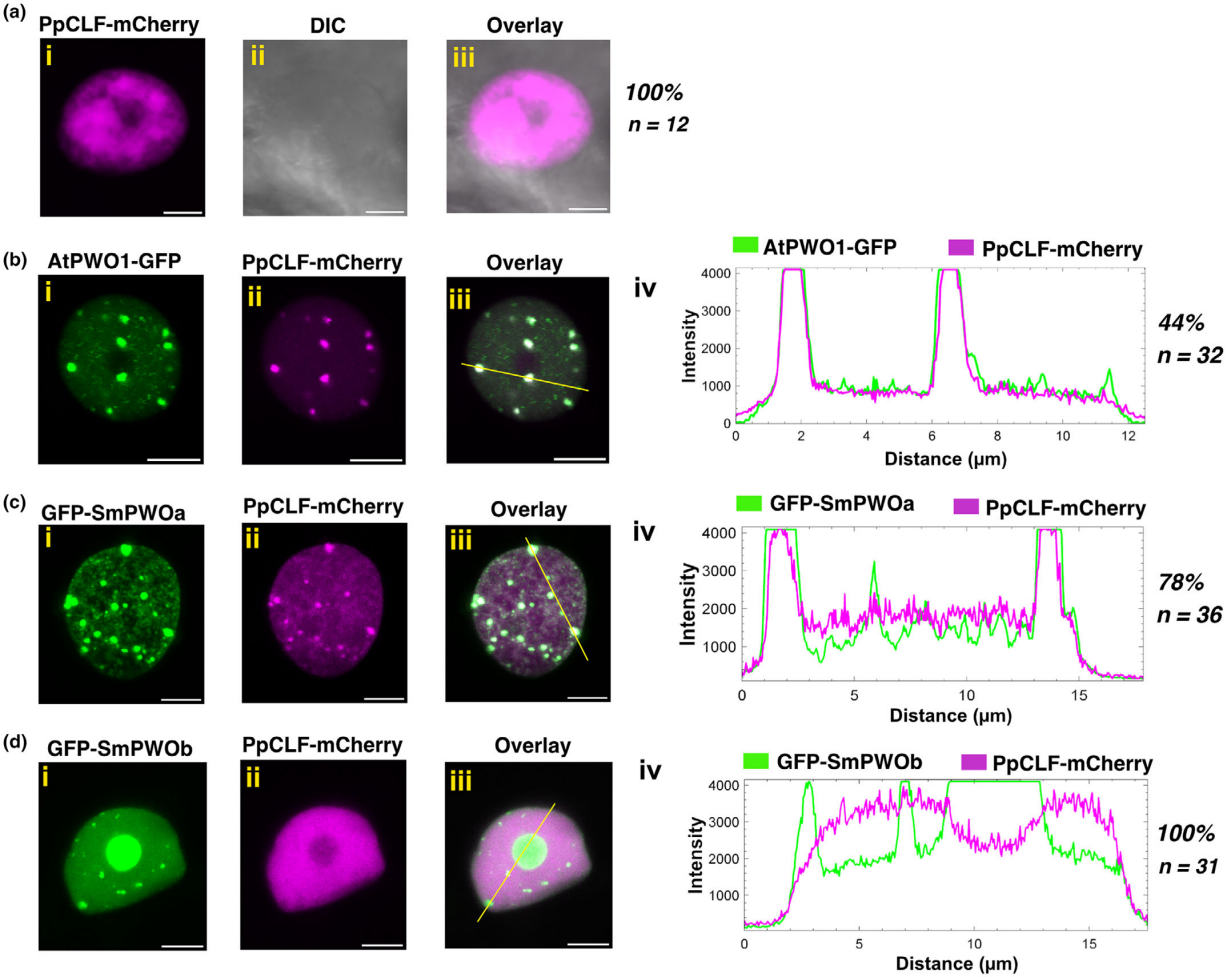
Colocalization of *Physcomitrium patens* (Pp) CLF with AtPWO1, SmPWOa, and SmPWOb in *Nicotiana benthamiana*. Representative confocal microscopy images showing nuclei of *N. benthamiana* leaf cells infiltrated with (ai–iii). *i35S*_*pro*_ *::PpCLF‐mCherry*, or co‐infiltrated with (bi–iii). *i35S*_*pro*_ *::AtPWO1‐GFP* and *i35S*_*pro*_ *::PpCLF‐mCherry*, (ci–iii) *i35Spro::GFP‐SmPWOa* and *i35S*_*pro*_ *::PpCLF‐mCherry* and (di–iii) *i35S*_*pro*_ *::GFP‐SmPWOb* and *i35S*_*pro*_ *::PpCLF‐mCherry*. (b–div) Profiles of mCherry and GFP fluorescence intensities along the yellow line in (b–diii). Percentage of nuclei with the observed pattern vs total number of analyzed nuclei (*n*) is indicated on the right. Bars, 5 μm.

Finally, we asked whether PWOs can tether PpCLF from *P. patens*, which lacks PWO proteins (Fig. 1a). When expressed alone, PpCLF‐mCherry diffusely localized in the nucleoplasm in non‐uniform patterns but did not form distinct speckles (Fig. 6a). Co‐expression of AtPWO1‐GFP (Fig. 6b) or GFP‐SmPWOa (Fig. 6c) and PpCLF‐mCherry resulted in the formation of PpCLF‐mCherry speckles in the nucleoplasm of 44% and 78% of nuclei, respectively. These speckles overlapped with AtPWO1‐GFP or GFP‐SmPWOa speckles (Figs 6b,c, S9A–C). By contrast, co‐expression of GFP‐SmPWOb and PpCLF‐mCherry resulted in neither the formation of PpCLF‐mCherry speckles in the nucleoplasm nor in PpCLF‐mCherry nucleolar signal (Fig. 6d).

### SmPWOa physically interacts with PRC2 catalytic subunits from Arabidopsis, *S. moellendorffii* and *P. patens*

**Fig. 7:**
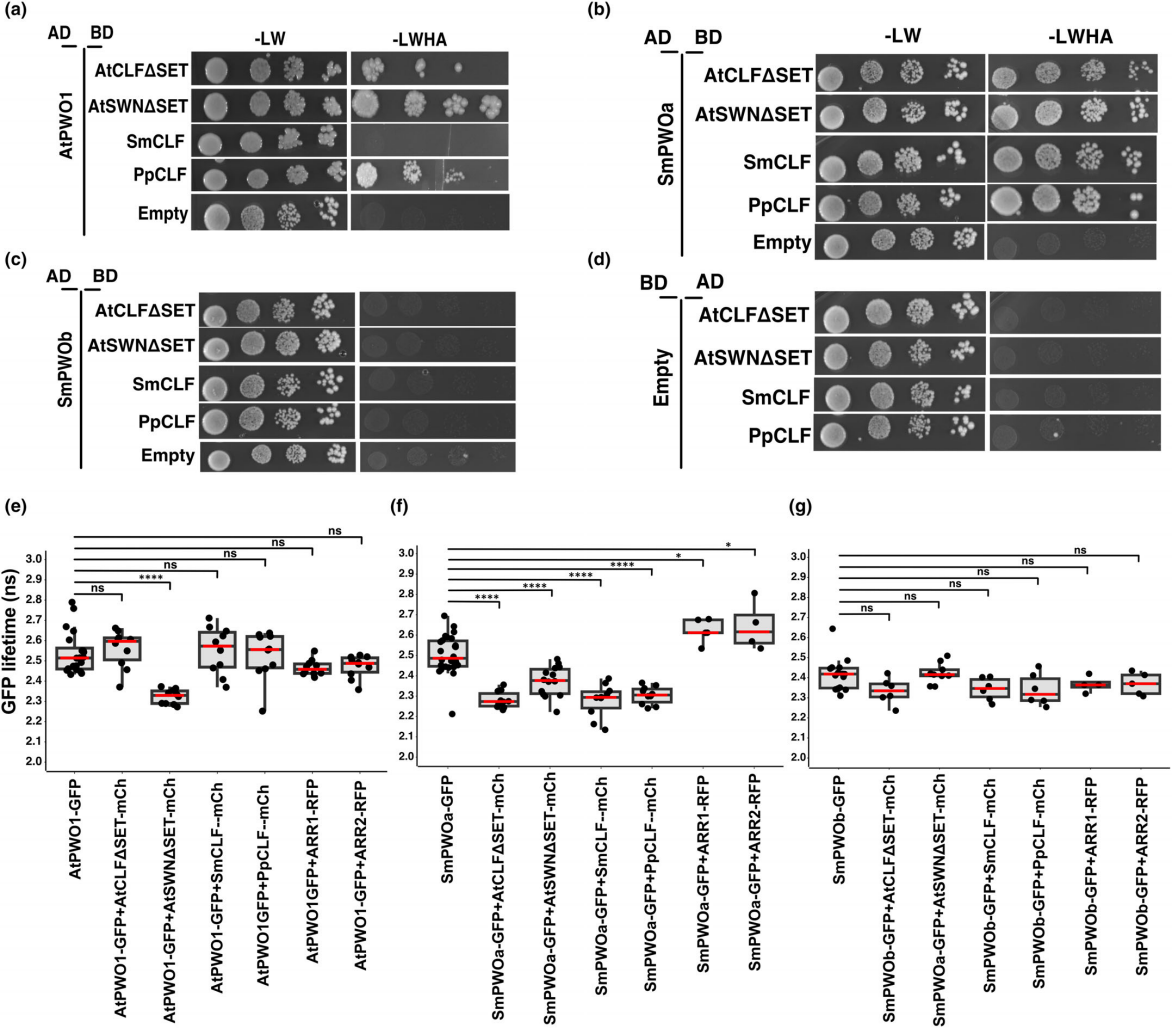
Protein–protein interactions of PWOs and PRC2 subunits. (a–c) Yeast two‐hybrid (Y2H) analyses of (a) AtPWO1, (b) SmPWOa, and (c) SmPWOb with AtCLFΔSET, AtSWNΔSET, PpCLF, and SmCLF. In the control panel (d), interactions for all combinations were assessed using an empty ‘BD’ plasmid. The interactions were evaluated by growing transformed yeast cells on non‐selective medium (−LW; lacking leucine and tryptophan) to facilitate plasmid co‐transformation and on selective medium (−LWAH; lacking leucine, tryptophan, adenine, and histidine) to activate the reporter gene. In this setup, ‘BD’ refers to the GAL4‐DNA‐binding domain, while ‘AD’ indicates the GAL4‐DNA‐activation domain fusion. Constructs containing CLF and SWN with SET domain deletions are denoted as ∆SET. (e–g) FLIM‐FRET analyses of GFP lifetime in *Nicotiana tabacum* nuclei co‐expressing *i35S*_*pro*_ *::mCherry‐AtCLFΔSET*, *i35S*_*pro*_ *::SmCLF‐mCherry*, and *i35S*_*pro*_ *::PpCLF‐mCherry* with (e) *i35S*_*pro*_ *::AtPWO1‐GFP*, (f) *i35S*_*pro*_ *::GFP‐SmPWOa* in speckles and (g) *i35S*_*pro*_ *::GFP‐SmPWOb* in nucleolus. Speckles or nucleolus were selected for measurements. For the negative control, each setup included *35S*_*pro*_ *::RFP‐ARR1*, *35S*_*pro*_ *::RFP‐ARR2* to co‐express those transcription factors with PWOs (SmPWOa, SmPWOb, AtPWO1). Boxes represent interquartile ranges, red horizontal lines represent the medians and whiskers represent SE; individual data points are shown. Statistical significance was determined using one‐way ANOVA, with multiple comparisons using Tukey's test. ****, *P* ≤ 0.0001; *, *P* ≤ 0.05; ‘ns’ indicates not significant with *P* > 0.05.

AtPWO1 and SmPWOa tether the PRC2 catalytic subunit CLF to their subnuclear compartments in *N. benthamiana* (Figs 4, 5, 6, S7–S9). Therefore, we asked whether this tethering involves direct physical interaction between PWOs and the CLF orthologs. Yeast two‐hybrid (Y2H) assays identified a physical interaction between AtPWO1 and AtSWN (Fig. 7a,d) (Hohenstatt *et al*., 2018). The AtPWO1‐AtCLF interaction, though weaker, was also confirmed (Fig. 7a,d). A weaker interaction is observed between AtPWO1 and PpCLF compared to AtPWO1‐AtSWN, while no significant interaction occurs between AtPWO1 and SmCLF (Fig. 7a,d). By contrast, SmPWOa interacts with the PRC2 catalytic subunit of *S. moellendorffii* (SmCLF), *P. patens* (PpCLF), and Arabidopsis (AtCLF/SWN) (Fig. 7b,d). A similar interaction for SmPWOb is not observed under high stringency (−LWHA) Y2H conditions (Fig. 7c,d), but under less stringent conditions (−LWH), a weaker interaction with PpCLF is detected (Fig. S10).

Förster resonance energy transfer (FRET) experiments measured by Fluorescence Lifetime Imaging Microscopy (FLIM) were performed to confirm these interactions (Fig. 7e–g). The FLIM‐FRET analyses were carried out either within nuclear speckles (AtPWO1, SmPWOa) or in the nucleolus (SmPWOb) of *N. tabacum* co‐expressing PWOs with the CLF orthologs from different species. Co‐expressing AtPWO1‐GFP with CLF orthologs (AtCLF‐, AtSWN‐, SmCLF‐, and PpCLF‐mCherry) resulted in a decrease in the AtPWO1‐GFP lifetime within nuclear speckles only when co‐expressed with AtSWN‐mCherry (Figs 7e, S11A). This supports the notion that AtPWO1 efficiently interacts with AtSWN but not with CLF from lower land plant models or not even with AtCLF. By contrast, co‐expressing GFP‐SmPWOa with AtCLF‐, AtSWN‐, SmCLF‐, and PpCLF‐mCherry resulted in a significant decrease in the fluorescence lifetime of GFP‐SmPWOa compared to sole expression of GFP‐SmPWOa, indicating direct physical interaction with all tested CLF orthologs (Figs 7f, S11B). However, co‐expression of GFP‐SmPWOb with the different CLF orthologs fused to mCherry resulted in no significant changes in the GFP‐SmPWOb lifetime, indicating lack of interaction under our experimental conditions (Figs 7g, S11C). As a negative control, we selected nuclear localized transcription factors ARABIDOPSIS RESPONSE REGULATOR1 (ARR1) and ARR2, which do not interact with PWOs, confirming the specificity of the interactions (Figs 7e–g, S11A–C). Overall, the Y2H and FLIM‐FRET analyses support the notion that AtPWO1 preferentially interacts with AtSWN rather than with AtCLF orthologs, as AtPWO1‐CLF interactions could not be confirmed using FLIM‐FRET. By contrast, SmPWOa displays a broader interaction capacity, showing direct binding to CLF orthologs from multiple species in both yeast and *N. tabacum*.

Arabidopsis AtPWO2 and AtPWO3 form a distinct sister Clade IV, separate from AtPWO1 (Fig. 1b). To assess whether CLF interaction and nucleoplasmic tethering are conserved features of Clade IV PWOs, we analyzed AtPWO2 as a representative member. In *N. benthamiana* leaf epidermal cells, AtPWO2‐GFP localized predominantly to the nucleoplasm, forming fewer and less distinct nuclear speckles than AtPWO1, and additionally exhibited a faint ring‐like structure at the nucleolus boundary, indicative of partial association with the perinucleolar region (Fig. S12A). AtPWO2‐GFP did not tether AtCLF to PWO2‐derived speckles (Fig. S12B) and showed no detectable interaction with AtCLF, AtSWN, SmCLF, or PpCLF in yeast two‐hybrid assays (Fig. S12C), in contrast to AtPWO1 (Figs 4b, S7A). A partial overlap between AtCLF and AtPWO2 at the nucleolus boundary was observed (Fig. S12B); however, considering the protein interaction results, this is unlikely to reflect a direct physical interaction between the two proteins. These results indicate that AtPWO2, a Clade IV PWO, may have distinct functions compared to Clade I (SmPWOs) and Clade III (AtPWO1), reflecting functional diversification within the PWO family.

### The conserved PWO C‐motif provides an interface for interactions with CLF orthologs

Based on the colocalization (Figs 3, 4, 5, 6) and interaction (Fig. 7) of PWO and CLF orthologs, we proceeded to investigate the interaction interface between them. Using Alphafold2‐Multimer (AF2‐M) (Evans *et al*., 2021; Homma *et al*., 2023, 2024; Ibrahim *et al*., 2023), we modeled the interactions between AtPWO1, AtSWN, and AtCLF. The interaction prediction for full‐length AtPWO1‐AtCLF, AtPWO1‐AtSWN or SmPWOa‐SmCLF did not detect a high‐confidence interaction interface, possibly due to the presence of IDRs in AtPWO1 (Figs S2, S13A–C; Table S8). However, when we systematically analyzed the interaction in a series of truncated PWO and CLF/SWN fragments using AF2‐M, we found that the central region of CLF/SWN, interacted with a truncated fragment of AtPWO1/SmPWOa lacking the PWWP domain (Fig. S14A,F,K). Specifically, the truncated fragments of AtCLF (166–364 aa) or AtSWN (166–364 aa) and AtPWO1 (377–769 aa) displayed local interactions (Fig. S14B–E,G–J). We also predicted a similar interaction surface for truncated SmCLF (166–364) and SmPWOa (377–619) (Fig. S14L–O). The C‐motif (Fig. 2) of AtPWO1 and SmPWOa was identified as the primary interface within these fragments for interaction with AtCLF (Fig. S14D,E), AtSWN (Fig. S14I,J) and SmCLF (Fig. S14N,O).

Given the conservation of the C‐motif across PWO phylogeny (Fig. S4), we modeled the interaction interfaces focusing on the PWO C‐motif and its interactions with CLF and SWN in representative species from different phylogenetic groups, including Arabidopsis (dicots), *Zea mays* (monocots), *Ceratopteris richardii* (Polypodiopsida), and *S. moellendorffii* (Lycopodopsida) (Fig. S15). The PWO C‐motif was predicted to be involved in the interaction with CLF orthologs across diverse land plant species (Fig. S15; Table S8).

**Fig. 8:**
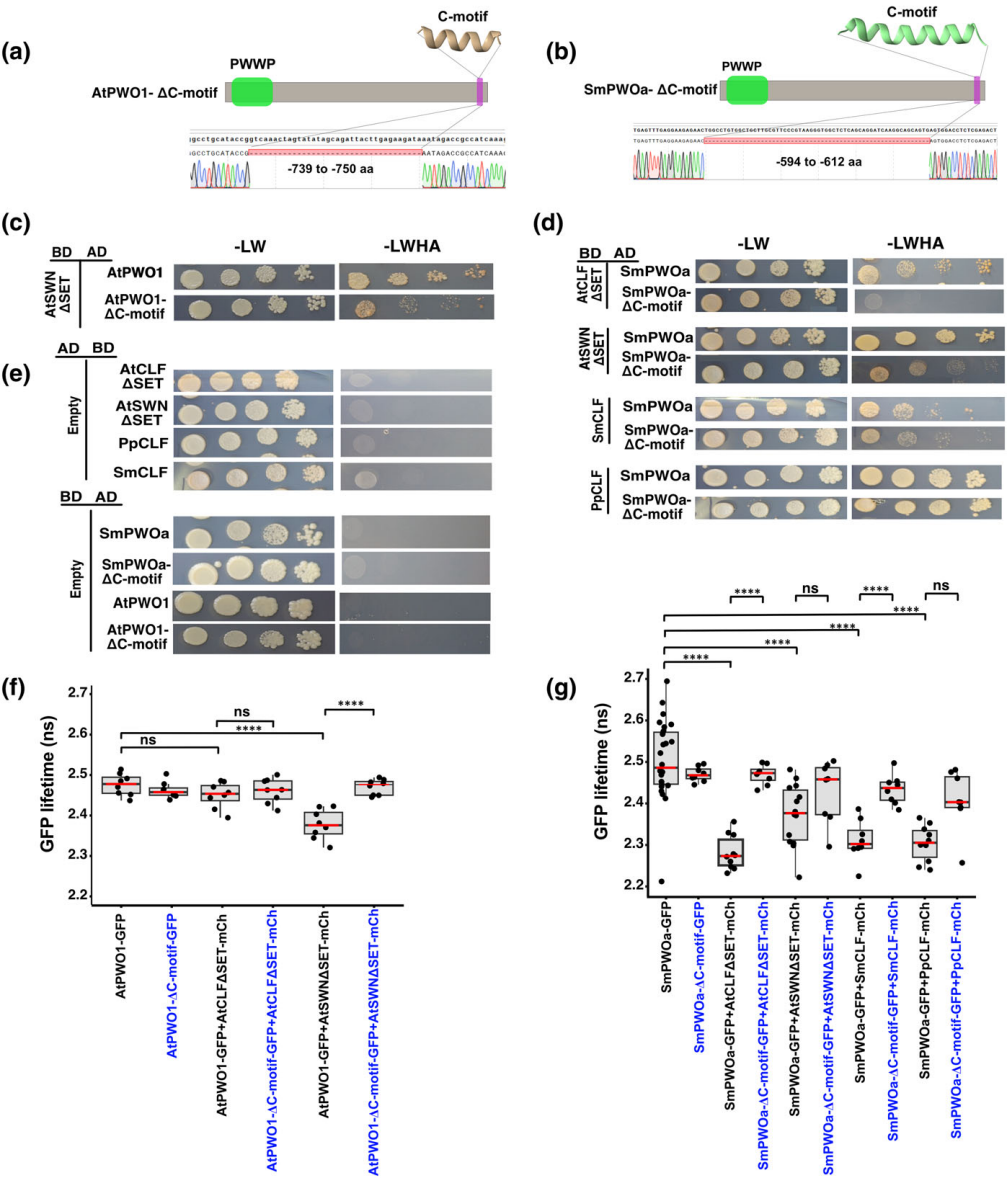
PWOs C‐motif engagement in interaction with PRC2 catalytic subunits. Schematic representation of the C‐motif deletion constructs of (a) AtPWO1 and (b) AtPWO1‐ΔC‐motif, used for Y2H and FLIM‐FRET analysis. Y2H analysis showing interactions of (c) AtPWO1 and AtPWO1‐ΔC‐motif (Δ739–750 aa) with AtSWNΔSET and (d) SmPWOa and SmPWOa‐ΔC‐motif (Δ594–612 aa) with AtSWNΔSET, AtCLFΔSET, SmCLF, and PpCLF, using ‐LW (non‐selective medium) and ‐LWAH (selective medium). (e) In the control panel for all combinations, interactions were assessed with an empty ‘BD’ or ‘AD’ plasmid. The ‘BD’ represents the GAL4 DNA‐binding domain, ‘AD’ represents the GAL4 DNA‐activation domain fusion and Δ represent for deletion of specific aa in the plasmid. (f, g) FLIM‐FRET analysis of GFP lifetime in *Nicotiana tabacum* nuclei co‐expressing (f) *i35S*_*pro*_ *::AtPWO1‐GFP* and *i35S*_*pro*_ *::AtPWO1‐ΔC‐motif‐GFP* and (g) *i35S*_*pro*_ *::GFP‐SmPWOa* and *i35S*_*pro*_ *::SmPWOa‐ΔC‐motif‐GFP with i35S*_*pro*_ *::mCherry‐AtCLFΔSET*, *i35S*_*pro*_ *::mCherry‐AtSWNΔSET*, *i35S*_*pro*_ *::SmCLF‐mCherry*, and *i35S*_*pro*_ *::PpCLF‐mCherry* within speckles. Boxes represent interquartile ranges, red horizontal lines represent the medians and whiskers represent SE; individual data points are shown. Statistical significance was determined using one‐way ANOVA with multiple comparisons using Tukey's test. ****, *P* ≤ 0.0001; ‘ns’ indicates not significant (*P* > 0.05). Blue highlighted combination represents C‐motif deleted constructs.

Truncated versions of AtPWO1‐ΔC‐motif (deletion of residues 739–750 aa) (Fig. 8a) and SmPWOa‐ΔC‐motif (deletion of residues 594–612 aa) (Fig. 8b) were used to assess the contribution of the C‐motif to interactions with the CLF orthologs. The Y2H results showed a reduced interaction between AtPWO1‐ΔC‐motif and AtSWNΔSET compared to the control (AtPWO1 with AtSWNΔSET) (Fig. 8c,e). SmPWOa‐ΔC‐motif with AtCLFΔSET showed a complete loss of interaction (Fig. 8d,e) and its interaction with AtSWNΔSET was reduced compared to the respective control setup using full‐length SmPWOa (Fig. 8d,e). Deletion of the C‐motif in SmPWOa did not considerably change the interaction between SmPWOa and SmCLF or PpCLF (Fig. 8d,e) in Y2H.

Co‐expression of AtPWO1‐ΔC‐motif‐GFP with AtSWN‐mCherry showed an increase in AtPWO1‐ΔC‐motif‐GFP fluorescence lifetime compared to AtPWO1‐GFP (WT) using FLIM‐FRET (Fig. 8f), supporting a lower binding affinity of the AtPWO1‐ΔC‐motif for AtSWN. Similarly, co‐expression of SmPWOa‐ΔC‐motif‐GFP with CLF (AtCLF, AtSWN, SmCLF, and PpCLF)‐mCherry resulted in a higher GFP fluorescence lifetime compared to full‐length SmPWOa‐GFP co‐expressed with CLF orthologs or SWN (Fig. 8g), supporting a reduced affinity of SmPWOa‐ΔC‐motif for CLF and SWN across species (Fig. 8g). Overall, these results indicate that the C‐motif participates in mediating PWO interactions with PRC2 components throughout plant evolution.

### *SmPWOa* partly complements the *pwo1‐1;pwo2‐2* double mutant phenotype in Arabidopsis

**Fig. 9:**
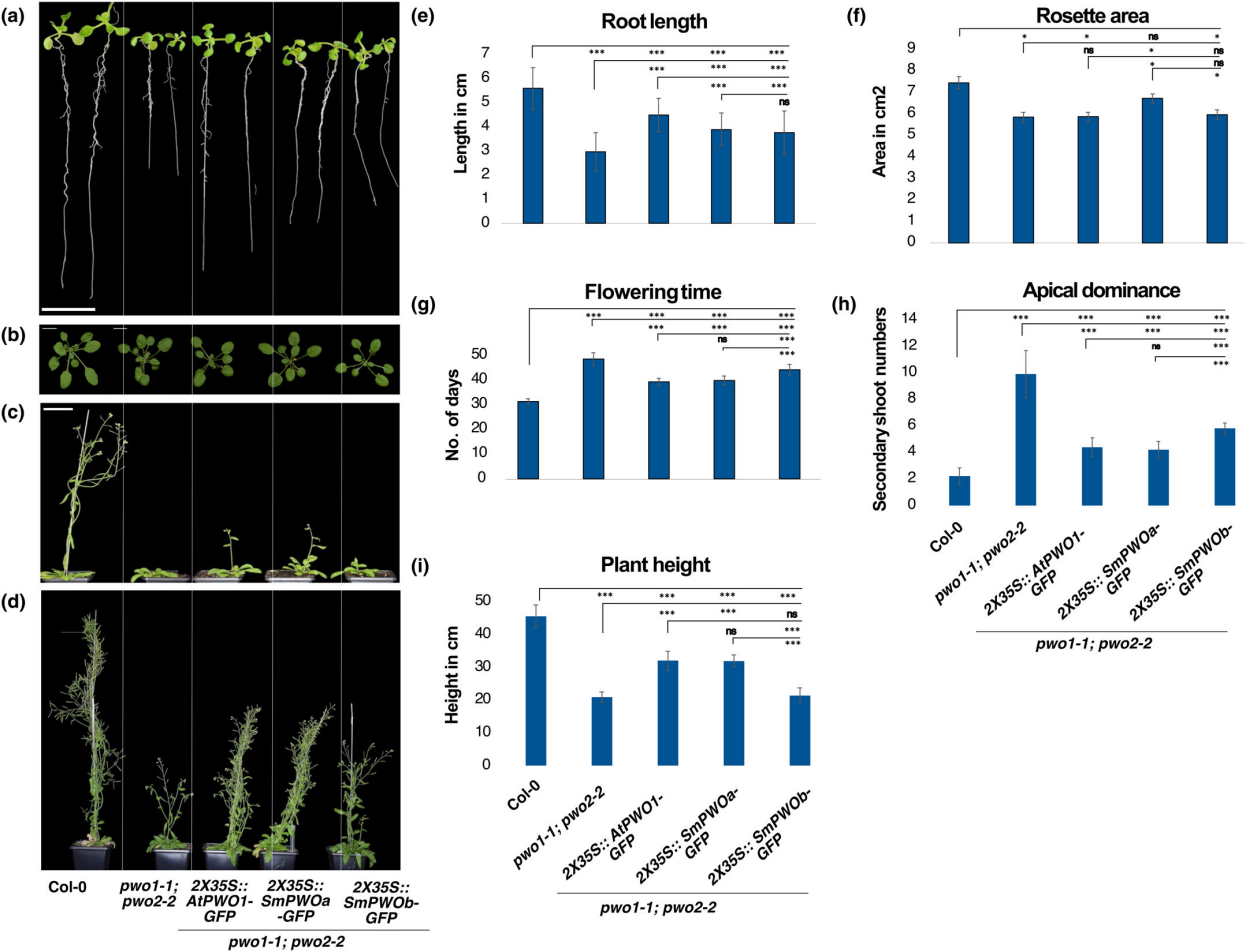
Phenotypic analyses of Arabidopsis *pwo1‐1;pwo2‐2* mutant lines overexpressing *AtPWO1*, *SmPWOa*, or *SmPWOb*. Representative images of (a) Root length phenotype in 10‐d‐old seedlings; (b) Rosette leaf area in 24‐d‐old plants; (c) Flowering time phenotype in 39‐d‐old plants; and (d) Plant height of 47‐d‐old plants for the genotypes Col‐0, *pwo1‐1;pwo2‐2*, *2x35S*_*pro*_ *::AtPWO1‐GFP*/*pwo1‐1;pwo2‐2*, *2x35S*_*pro*_ *::SmPWOa‐GFP*/*pwo1‐1;pwo2‐2*, and *2x35S*_*pro*_ *::SmPWOb‐GFP*/*pwo1‐1;pwo2‐2* lines. (e) Root length (*n* = 28); (f) Rosette leaf area (*n* = 10); (g) Flowering time (*n* = 10); (h) Rosette leaves number at flowering time (*n* = 10); (i) Plant height (*n* = 10). Error bars correspond to ±SD. Asterisks represent *P*‐values: ***, *P* ≤ 0.001; *, *P* ≤ 0.05. The two‐tailed Student's *t*‐test was applied to calculate the significance level. Bars, 5 μm. "ns" indicates not significant (*P* > 0.05).

We expressed *AtPWO1‐GFP*, *SmPWOa‐GFP* or *SmPWOb‐GFP* in the Arabidopsis *pwo1‐1;pwo2‐2* double mutant background to determine their potential for restoring a WT‐like phenotype. First, we confirmed that *AtPWO1‐GFP* can substantially complement several developmental phenotypes in *pwo1‐1; pwo2‐2* plants (Figs 9, S16F). Interestingly, *SmPWOa‐GFP* could fully or partially complement nearly all the developmental defects of the *pwo1‐1;pwo2‐2* mutant (i.e. root length, rosette area, plant height, nuclear size, and flowering time), similarly to (and sometimes exceeding) AtPWO1, while *SmPWOb‐GFP* only partially complemented some of the traits (Figs 9a–j, S16F). For example, *pwo1‐1;pwo2‐2* lines complemented with *AtPWO1* and *SmPWOs* showed an increase in root length compared to *pwo1‐1;pwo2‐2* double mutant. However, neither of these contracts fully complement this trait (Fig. 9a,e). *pwo1‐1;pwo2‐2*, *AtPWO1‐GFP and SmPWOb‐GFP* displayed reduced rosette size (total leaf area) compared to WT, while rosette size was restored in *SmPWOa‐GFP* (Fig. 9b,f). A delay in flowering is observed in *pwo1‐1;pwo2‐2* double mutants, which was partially restored by *AtPWO1‐GFP* or *SmPWOa‐GFP*, but significantly less by *SmPWOb*‐*GFP* (Fig. 9c,g). While *pwo1‐1;pwo2‐2* double mutants showed reduced height, *AtPWO1* and *SmPWOa‐GFP* plants were significantly taller than the mutant plants (Fig. 9d,i). The loss of apical dominance in *pwo1‐1;pwo2‐2* double mutant was partially complemented by all constructs (Fig. 9h). Similarly, *SmPWOa‐GFP* partially restored reproductive defects in *pwo1‐1;pwo2‐2*, including the reduced silique number per plant and seed number per silique, whereas *SmPWOb‐GFP* only partially complemented the silique number phenotype (Fig. S16A,B). Previously, *pwo1‐1* mutants were shown to exhibit a reduced nuclear area and an increased circularity index (Mikulski *et al*., 2019). A similar phenotype was confirmed for *pwo1‐1;pwo2‐2* (Fig. S16C–E). Nuclear area of *pwo1‐1;pwo2‐2* was complemented by both *SmPWO* constructs, while circularity index was fully restored to WT level only by *SmPWOa‐GFP* (Fig. S16C–E). Finally, we examined whether *SmPWOa* and *SmPWOb* could rescue the developmental lethality of the *pwo1‐1;pwo2‐2;pwo3‐1* triple mutant. *pwo1‐1;pwo2‐2 SmPWOa‐GFP* and *pwo1‐1;pwo2‐2 SmPWOb‐GFP* transgenic lines were crossed with *pwo3‐1*, and F3 progeny carrying the transgenes in the triple mutant background were analyzed. Although *SmPWOa*, and, to a lesser extent *SmPWOb*, partially complemented several phenotypic traits in *pwo1‐1;pwo2‐2* double mutant background (Figs 9a–j, S16F), neither was able to rescue seedling developmental lethality in the triple mutant background.

Previous transcriptomic analyses demonstrated that PWO proteins contribute to the regulation of gene expression and influence the levels of the PRC2‐associated histone modification H3K27me3 (Hohenstatt *et al*., 2018; Mikulski *et al*., 2019; Zheng *et al*., 2023; Yang *et al*., 2024). Based on these findings, we selected a subset of genes previously shown to be misregulated in the *pwo1‐1;pwo2‐2* mutant (Zheng *et al*., 2023) and analyzed their expression in the *SmPWOa/b‐GFP*/*pwo1‐1;pwo2‐2* lines. Expression of *ACTIN1* (*ACT1*) and *SHOOT MERISTEMLESS* (*STM*) was restored to wild‐type levels by *SmPWOa‐GFP*, whereas *SAMBA* expression was increased. By contrast, expression of *GAMETOPHYTE DEFECTIVE 1* (*GAF1*) and *SAMBA* was restored only in *SmPWOb‐GFP* lines (Fig. S17A–D).

Next, we assessed whether *SmPWOa‐GFP* and *SmPWOb‐GFP* could restore H3K27me3 enrichment at selected AtPWO1 target loci in the *pwo1‐1;pwo2‐2* mutant background (Zheng *et al*., 2023; Yang *et al*., 2024). Chromatin immunoprecipitation analyses revealed locus‐specific effects (Fig. S17E). H3K27me3 levels at *METALLOENDOPROTEINASE 3‐MMP* (*AT3‐MMP*) and *GAMETOPHYTE DEFECTIVE 1* (*GAF1*) were comparable to wild‐type levels across all genotypes. By contrast, *WRKY48*, *HECATE1* (*HEC1*), and *UDP‐GLYCOSYLTRANSFERASE 76E7* (*UGT76E7*) exhibited reduced H3K27me3 enrichment in *pwo1‐1;pwo2‐2* and *SmPWOb‐GFP* complementation lines, whereas *SmPWOa‐GFP* restored H3K27me3 levels to those observed in wild‐type. Conversely, *FLOWERING BHLH 3* (*FBH3*) showed increased H3K27me3 enrichment in *pwo1‐1;pwo2‐2*, while both *SmPWOa‐GFP* and *SmPWOb‐GFP* restored H3K27me3 levels to those of wild‐type. Finally, H3K27me3 enrichment at *CYP94C1* was similar to wild‐type in *pwo1‐1;pwo2‐2* and *SmPWOa* lines but was reduced in *SmPWOb* lines (Fig. S17E). These results together show that SmPWOa or SmPWOb can partially complement the lack of AtPWOs activity in Arabidopsis. However, SmPWOa consistently shows a higher potential for phenotypic and molecular complementation, suggesting a higher level of functional conservation.

## Discussion

### PWOs emerged from lycophytes and are well conserved in vascular plants

The transition from aquatic to terrestrial habitats is connected to the emergence of key new traits that enabled the plants to endure sudden environmental changes (Spencer *et al*., 2021; Woudenberg *et al*., 2022). These traits were linked with genetic variation that facilitated the emergence of new genes. Such genetic innovations primarily involved transcriptional regulators, which are typically associated with biotic and abiotic stress responses (Donoghue *et al*., 2021). PWOs emerged in lycophytes, the early‐diverged group of vascular plants (Fig. 1a). Therefore, it is tempting to speculate that the appearance of PWOs at this critical point in plant evolution may have been necessary for land plants to acquire new developmental or adaptive traits required for survival in terrestrial environments. The presence of PWOs in distinct clades (Fig. 1b) highlights both evolutionary conservation and divergence, which may lead to sub‐ and neofunctionalization, facilitating the evolution of novel traits (Ohno, 1970; Force *et al*., 1999; Zalewski *et al*., 2013). Two major separate clades of PWO proteins are found in seed plants, represented by Arabidopsis AtPWO1 and AtPWO2/AtPWO3 (Fig. 1b). While AtPWO1 interacts with the catalytic subunits of PRC2 and tethers CLF to prominent subnuclear speckles, AtPWO2 forms very few speckles and predominantly accumulates at the nucleolar periphery. Consistently, we could not confirm such interactions or comparable CLF tethering for AtPWO2 (Fig. S12), supporting functional diversification between the two PWO clades in Arabidopsis. Similarly, two independently emerged PWO clades are found in Lycopodiopsida, represented by *S. moellendorfii* SmPWOa and SmPWOb (Fig. 1b). Interestingly, SmPWOa and SmPWOb display distinct nuclear localization and protein–protein binding characteristics (Figs 3, 4, 5, 6, 7), supporting at least partial divergence. The conservation of PWO proteins in vascular plants highlights their functional importance and suggests their essentiality. Indeed, full absence of PWOs in Arabidopsis is seedling lethal, suggesting a prime role for PWOs in plant development and a partial redundancy of AtPWO1‐3 (Hohenstatt *et al*., 2018). Whether redundancy between PWOs may exist in lycophytes needs to be determined in the future. Nevertheless, considering the localization of SmPWOa (Fig. 3a,d) and SmPWOb (Fig. 3b,e) within the nucleus, it is possible that SmPWOs may function together or exhibit overlapping functions. Therefore, understanding the functions of SmPWOs in *S. moellendorfii* and how they contribute to the acquisition of new traits for land plant evolution are key questions to address in the future.

### PWOs associate with PRC2 since their emergence in lycophytes and have conserved functions in plant development

A distinct feature of the PWO proteins is the presence of highly IDRs (Fig. S2). IDRs function as protein assemblers, providing large binding interfaces that scaffold multiple partners to facilitate higher‐order protein complex formation. Proteins containing IDRs often facilitate both stable and transient protein–protein interactions, play a crucial role in transcription and chromatin regulation, mediate stress responses, and contribute to development (Sun *et al*., 2013; Salladini *et al*., 2020; Cermakova & Hodges, 2023; Hsiao, 2024; Gupta *et al*., 2025; Miao & Chong, 2025). Therefore, we propose that the presence of IDRs in PWO proteins across multiple evolutionary groups may confer conserved PWO functions, such as its ability to interact with other chromatin regulators (Tan *et al*., 2018; Zheng *et al*., 2023; Godwin *et al*., 2024). IDRs are also known to facilitate phase separation by selectively partitioning proteins in the subnuclear space through liquid–liquid phase separation (LLPS), forming different types of nuclear speckles (Cermakova & Hodges, 2023). These nuclear bodies (NBs) are often associated with specific genomic loci and exhibit a dynamic nature in response to different environmental stimuli, including acclimation to stress conditions and immune responses in plants (Wang & Gu, 2022; Solis‐Miranda *et al*., 2023). As PWOs form varying amounts of nuclear speckles/NBs in the nucleoplasm (Figs 3, 4, 5, 6, S12), it will be interesting to determine their possible dynamics in stress conditions and to address a possible role of PWOs in stress responses, as previously suggested (Hohenstatt *et al*., 2018; Mikulski *et al*., 2019). It will be of particular interest to determine whether PWOs may confer resistance to environmental challenges imposed especially on land plants.

In Arabidopsis, PWOs interact with key chromatin regulatory complexes involved in gene activation (e.g. histone acetyl transferases and deubiquitinases) or repression (e.g. PRC2) (Hohenstatt *et al*., 2018; Tan *et al*., 2018; Zheng *et al*., 2023; Godwin *et al*., 2024). The PWO‐CLF interaction is conserved in the orthologs SmPWOa and SmCLF (Fig. 7b,f). Interestingly, SmPWOa interacts with CLF of *P. patens* (PpCLF) (Fig. 7b,f), where PWO proteins are absent. This indicates that PWO emerged to interact with a conserved CLF interaction surface rather than vice versa. Unlike PWOs, most known PWO interactors, such as PRC2 (Huang *et al*., 2017; Sharaf *et al*., 2022; de Potter *et al*., 2023) and UBP5 (Zheng *et al*., 2023; Godwin *et al*., 2024), are evolutionarily conserved across the green lineage. Similarly, the PWO1‐interacting TRB proteins are also conserved from bryophytes to flowering plants (Kusová *et al*., 2023; Amiard *et al*., 2024). The emergence of PWOs in lycophytes may also correlate with the shift in H3K27me3 targeting from TEs to protein‐coding genes (Hisanaga *et al*., 2023). Interestingly, AtPWO1 exhibits a robust interaction with AtSWN (Fig. 7a,e, Hohenstatt *et al*., 2018), while its interaction with basic land plant CLFs (SmCLF, PpCLF) was weaker or insignificant (Fig. 7a,e). This specificity may have originated through specialization of the higher‐plant AtPWO1 clade toward SWN, which emerged only in angiosperms. By contrast, ancestral PWO proteins may have retained the ability to interact with CLF orthologs across species, consistent with CLF being the most ancestral PRC2 catalytic subunit (Huang *et al*., 2017). One of the key features of AtPWO1 and SmPWOs observed upon co‐expression with CLF proteins in *N. benthamiana* was the tethering of CLF to PWO‐derived speckles (Figs 4, 5, 6), including for those cases in which we could not confirm a direct interaction (e.g. AtPWO1 tethering Sm/PsCLF to speckles). This could indicate that in our plant heterologous system additional factors might also contribute to protein re‐localization. Therefore, PWOs may play a crucial role in spatially organizing CLF within the nucleus, possibly affecting its function in chromatin modulation. Together, this suggests that CLF orthologs possess a conserved region for PWO interaction, even in species lacking PWOs. It remains unclear whether this conservation is associated with the presence of other accessory proteins that may substitute for PWOs in *P. patens* or whether other conserved proteins exist across all land plants that interact with CLF through the same interaction surface as PWOs.

The nucleolar localization of SmPWOb (Figs 3, 4, 5, 6) and the peri‐nucleolar distribution of AtPWO2 (Fig. S12A,B) suggest that some PWO orthologs may have nucleolus‐associated functions. Similarly, nucleolar localization is observed for TRB proteins, which directly interact with PRC2 (Zhou *et al*., 2018), and are components of the PEAT complex (Zheng *et al*., 2023). In the nucleolus, TRB proteins interact with the components of the telomerase biogenesis complex hypothesized to partake in telomerase RNP biogenesis (Schořová *et al*., 2019) but beyond this finding, the nucleolar protein interaction network or functional significance of nucleolar targeting of these proteins remain unclear.

In addition to the PWWP domain (Fig. S1), we identified a conserved short helical structure (C‐motif) located at the C‐terminal region in all PWO proteins across plant evolutionary clades (Figs 2, S4, S5). The C‐motifs that contain an array of hydrophobic amino acids may facilitate interactions with other proteins or help stabilize the structure (Gibbons *et al*., 2005; Jernigan *et al*., 2022). Structural predictions indicate that the PWO C‐motif may interact with CLF orthologs in different species throughout evolution, suggesting that the C‐motif may serve as a conserved component for CLF ortholog binding. Accordingly, deletion of the C‐motif significantly reduces the AtPWO1‐AtSWN interaction and the interaction of SmPWOa with all tested CLF orthologs (Fig. 8). Therefore, we propose that the C‐motif of the PWO proteins plays an important role in mediating the evolutionarily conserved direct interaction between PWOs and the PRC2 catalytic subunit. It will be interesting to determine whether the function of the C‐motif may extend to protein components of other chromatin modifiers and to what extent the co‐evolution of the PWWP domain with the C‐motif may define the conservation of PWO function in distinct plant species.

Overall, we have shown that the PWO‐PRC2 interaction is evolutionarily conserved (Fig. 7a,b,e,f). However, the biological relevance of PWO interaction with PRC2 throughout evolution and the significance of its emergence in lycophytes remain unresolved. Recent evidence demonstrated that PWO1 binds to the boundaries of H3K27me3‐CDs and plays a crucial role in maintaining these boundaries. This function contributes to the 3D nuclear organization and the repressed state of these CDs (Yang *et al*., 2024). Although the impact of the PWO1‐PRC2 interaction on this activity is still unknown, it has been suggested that PWOs could help to phase‐separate PRC2 to reinforce its CD boundaries (Yang *et al*., 2024). Hence, it is tempting to speculate that the appearance of PWOs may have contributed to the relocation of PRC2 and H3K27me3. Whether this also biased PRC2 targeting and H3K27me3 distribution away from TEs toward protein‐coding genes during the evolution of land plants (Hisanaga *et al*., 2023) is an attractive hypothesis worth exploring in the future.

Despite an evolutionary separation of 429 million years (Kumar *et al*., 2017), the partial complementation of the Arabidopsis *pwo1;pwo2* double mutant by *SmPWOs* suggests a certain degree of functional conservation. *SmPWOa‐GFP* showed a similar level of functional complementation for developmental defects of *pwo1‐1;pwo2‐2* as *AtPWO1‐GFP* (Figs 9, S16), but it failed to complement the lethality of the *pwo1‐1;pwo2‐2;pwo3‐1* triple mutant. The partial complementation may reflect the differences in functions of AtPWO1 and AtPWO2, which is also supported by the different nuclear localization of AtPWO1 and AtPWO2 and their association with PRC2 subunits (Figs 4, 7, S12). Similarly, the weaker complementation of *pwo1‐1;pwo2‐2* developmental (Figs 9, S16), gene expression and H3K27me3 (Fig. S17) defects by SmPWOb compared to SmPWOa indicates functional diversification of the two SmPWO proteins. Likewise, AtPWO1 and AtPWO2 may differ due to their distinct intranuclear localization (Figs 3, S12A) as well as their differing abilities to interact with CLF orthologs (Figs 7, S12C). Differences in PWO functions, both between and within species, may be related to the low level of conservation in the IDR‐containing region of PWO proteins. Differences in these regions are likely to significantly influence PWO functions through protein–protein interaction networks specific to the individual PWOs, as most PWO1 interactors (ARIDs, EPCR1, TRB1‐2, and UBP5) primarily bind to PWOs' IDRs in Arabidopsis (Zheng *et al*., 2023). Considering the similar features of AtPWO1 and SmPWOa, and possibly AtPWO2 and SmPWOb, in terms of nuclear localization and potential interactions with PRC2 subunits, it is tempting to speculate that the functional specialization of lycophyte and flowering plant PWOs may have followed a similar trajectory, driven by comparable functional requirements. Despite the differences between the different PWO subclades, lycophyte and flowering plant PWOs seem to retain key molecular functions that contribute to plant development, gene regulation and nuclear organization. It will be intriguing to further investigate the level of conservation of the PWO‐interacting protein network across evolution and to explore how PWOs contribute to establishing novel interactions or to strengthening existing ones to reshape the chromatin landscape.

## Competing interests

None declared.

## Author contributions

Ahamed Khan, IM, and SF conceptualized the experimental approach and designed the methodology. Ahamed Khan performed most of the molecular experiments, including construct generation, protein localization, transgenic plant generation, confocal microscopy analyses, and data visualization, as well as manuscript writing and revision. SH conducted genotyping and phenotyping of transgenic lines as well as gene expression analyses and ChIP‐qPCR for the transgene complementation experiments. AS and Ahamed Khan conducted the phylogenetic analyses. Alžbeta Kusová and JS carried out the FLIM‐FRET experiments. CJ and Ahamed Khan performed the nuclear morphology analysis. Ahamed Khan and MR performed and interpreted AF modeling. PPS, JH, and DS contributed to substantially revising and improving the manuscript. All authors contributed to the interpretation of the results. Ahamed Khan, IM, and SF wrote and revised the manuscript.

## Disclaimer

The New Phytologist Foundation remains neutral with regard to jurisdictional claims in maps and in any institutional affiliations.

## Supporting information


**Fig. S1** Multiple Sequence Alignment of the conserved PWWP domain across plant evolution.
**Fig. S2** Bioinformatic analysis of Intrinsically Disordered Regions in PWO proteins.
**Fig. S3** AlphaFold2 structure prediction of *Arabidopsis thaliana* and *Selaginella moellendorfii* PWO protein PWWP domains.
**Fig. S4** Alphafold2‐based structure prediction of the C‐motif in PWO proteins across representative species with PWO clades.
**Fig. S5** Amino acid multiple sequence alignments of PWO C‐terminal region including C‐motif.
**Fig. S6** Co‐infiltration of PWOs (AtPWO1, SmPWOa, SmPWOb) with empty mCherry.
**Fig. S7** Colocalization of SmPWOs and AtWPO1 with AtCLF in *Nicotiana benthamiana*.
**Fig. S8** Localization and colocalization of SmCLF with AtPWO1 and SmPWOa in *Nicotiana benthamiana*.
**Fig. S9** Colocalization of AtPWO1 and SmPWOa with *Physcomitrium patens* (Pp)CLF in *Nicotiana benthamiana*.
**Fig. S10** Yeast two‐hybrid assays for SmPWOb and PRC2 catalytic subunit interactions.
**Fig. S11** FLIM‐FRET confocal microscopy images for PWOs and CLF orthologs.
**Fig. S12** PWO2 localization, colocalization, and interaction with CLF.
**Fig. 13** Alphafold2‐Multimer‐based prediction of interaction surfaces between PWOs and CLF or SWN in *Arabidopsis thaliana* and *Selaginella moellendorffii*.
**Fig. S14** AF2‐M‐based prediction of the interaction surface between truncated PWOs and CLF/SWN.
**Fig. S15** AF2‐M prediction of interaction surfaces between PWOs and PRC2 catalytic subunit across plant species.
**Fig. S16** Role of PWOs in Arabidopsis development and nuclear morphology.
**Fig. S17** RT‐qPCR analyses of genes misregulated in *pwo1‐1;pwo2‐2* and levels of H3K27me3 at PWO1 targets in *pwo1;pwo2* and complemented lines.

**Table S1** List of primers used in the study.
**Table S2** Identified PWO orthologs.
**Table S3** Data for the phylogenetic tree of PWO orthologs.
**Table S4** NLS prediction of PWO proteins using DeepLoc‐2.0.
**Table S5** Full‐length sequences of PWO proteins highlight the SWWP motif in purple and the C‐motif in red.
**Table S6** RMSD (Ca) scores of PWWP domains for AtPWOs and SmPWOs when superimposed to AtPWO1.
**Table S7** RMSD (Ca) scores of C‐motifs for Clade I‐IV when superimposed to AtPWO1.
**Table S8** pTM and ipTM scores for all AF2‐M predicted structures and interactions.
Please note: Wiley is not responsible for the content or functionality of any Supporting Information supplied by the authors. Any queries (other than missing material) should be directed to the *New Phytologist* Central Office.

## Data Availability

Sequence information for the genes used in this study can be found in the GenBank/EMBL data libraries under the following accession numbers: *AT3G03140* (*PWO1*), *AT4G02020* (*AtSWN*), *AT2G23380* (*AtCLF*), *PZ189264* (*SmPWOa*), *PZ189265* (*SmPWOb*), *XP_002987466.1* (*SmCLF*), *AB472766.1* (*PpCLF*), *AT2G37620* (*ACT1*), *AT1G62360* (*STM*), *AT5G59980* (*GAF1*), *AT1G32310* (*SAMBA*), *AT4G34270* (*TIP41*), *AT1G24140* (*AT3‐MMP*), *AT5G49520* (*WRKY48*), *AT5G38040* (*UGT76E7*), *AT5G67060* (*HEC1*), *AT2G27690* (*CYP94C1*), *and AT1G51140* (*FBH3*).
