# Supplementary material for: PWO proteins are associated with PRC2 since their emergence in vascular plants
Source: New Phytol. 2026 Apr 20;250(6):4002–21. doi: 10.1111/nph.71172 (PMC13193366; doi:10.1111/nph.71172)
Supplement: Supplementary file 1 — Fig. S1 Multiple Sequence Alignment of the conserved PWWP domain across plant evolution. Fig. S2 Bioinformatic analysis of Intrinsically Disordered Regions in PWO proteins. Fig. S3 AlphaFold2 structure prediction of Arabidopsis thaliana and Selaginella moellendorfii PWO protein PWWP domains. Fig. S4 Alphafold2‐based structure prediction of the C‐motif in PWO proteins across representative species with PWO clades. Fig. S5 Amino acid multiple sequence alignments of PWO C‐terminal region including C‐motif. Fig. S6 Co‐infiltration of PWOs (AtPWO1, SmPWOa, SmPWOb) with empty mCherry. Fig. S7 Colocalization of SmPWOs and AtWPO1 with AtCLF in Nicotiana benthamiana. Fig. S8 Localization and colocalization of SmCLF with AtPWO1 and SmPWOa in Nicotiana benthamiana. Fig. S9 Colocalization of AtPWO1 and SmPWOa with Physcomitrium patens (Pp)CLF in Nicotiana benthamiana. Fig. S10 Yeast two‐hybrid assays for SmPWOb and PRC2 catalytic subunit interactions. Fig. S11 FLIM‐FRET confocal microscopy images for PWOs and CLF orthologs. Fig. S12 PWO2 localization, colocalization, and interaction with CLF. Fig. 13 Alphafold2‐Multimer‐based prediction of interaction surfaces between PWOs and CLF or SWN in Arabidopsis thaliana and Selaginella moellendorffii. Fig. S14 AF2‐M‐based prediction of the interaction surface between truncated PWOs and CLF/SWN. Fig. S15 AF2‐M prediction of interaction surfaces between PWOs and PRC2 catalytic subunit across plant species. Fig. S16 Role of PWOs in Arabidopsis development and nuclear morphology. Fig. S17 RT‐qPCR analyses of genes misregulated in pwo1‐1;pwo2‐2 and levels of H3K27me3 at PWO1 targets in pwo1;pwo2 and complemented lines. [file NPH-250-4002-s002.docx]

**New Phytologist Supporting Information Word file.**

**Article title:** PWO proteins are associated with PRC2 since their emergence in vascular plants

**Authors:** Ahamed Khan, Saqlain Haider, Abdoallah Sharaf, Alžbeta Kusová, Jan Skalák, Claire Jourdain, Martin Rennie, Petra Procházková Schrumpfová, Jan Hejátko, Daniel Schubert, Sara Farrona, Iva Mozgová

**Article acceptance date:** 16 March 2026


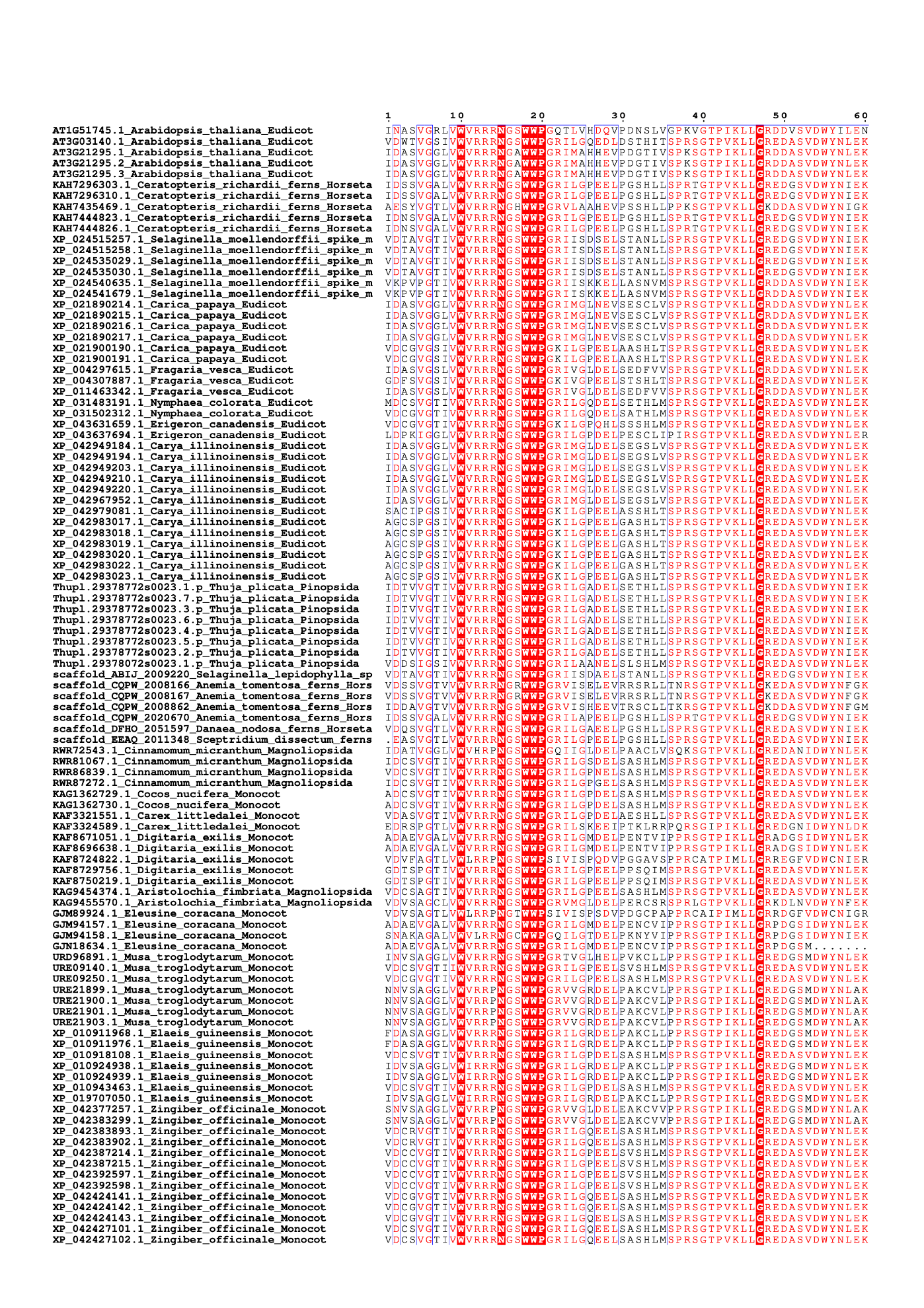


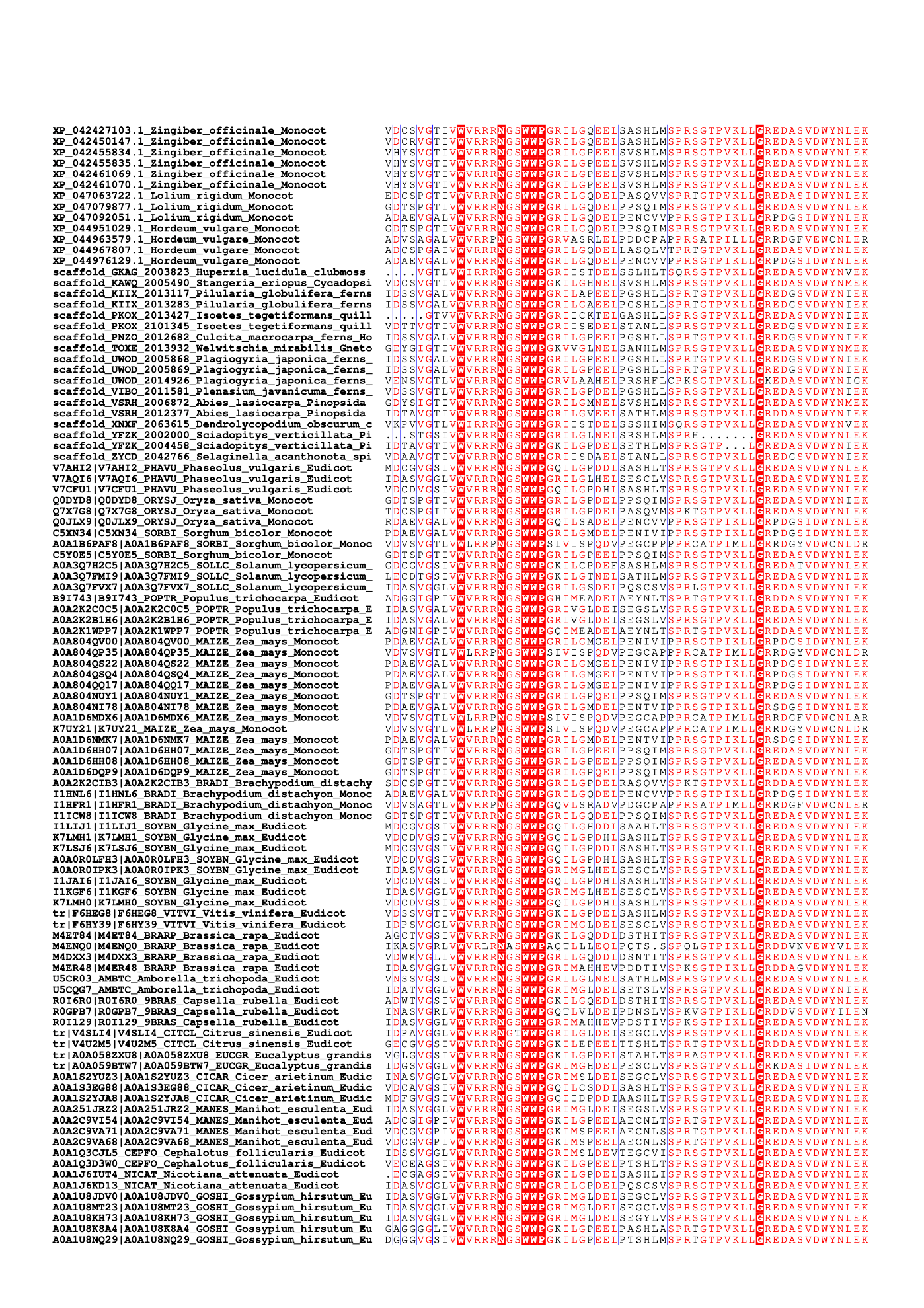


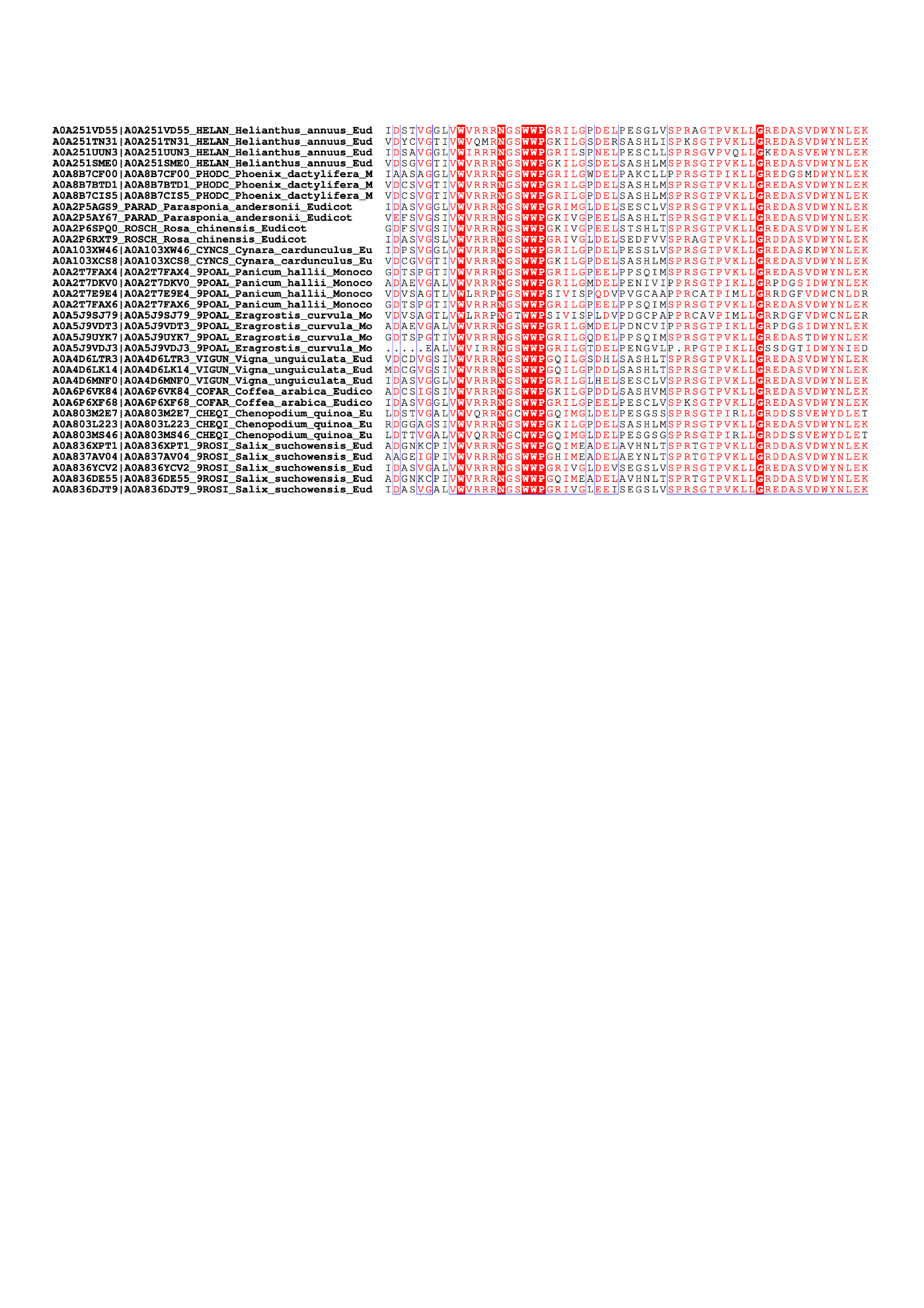


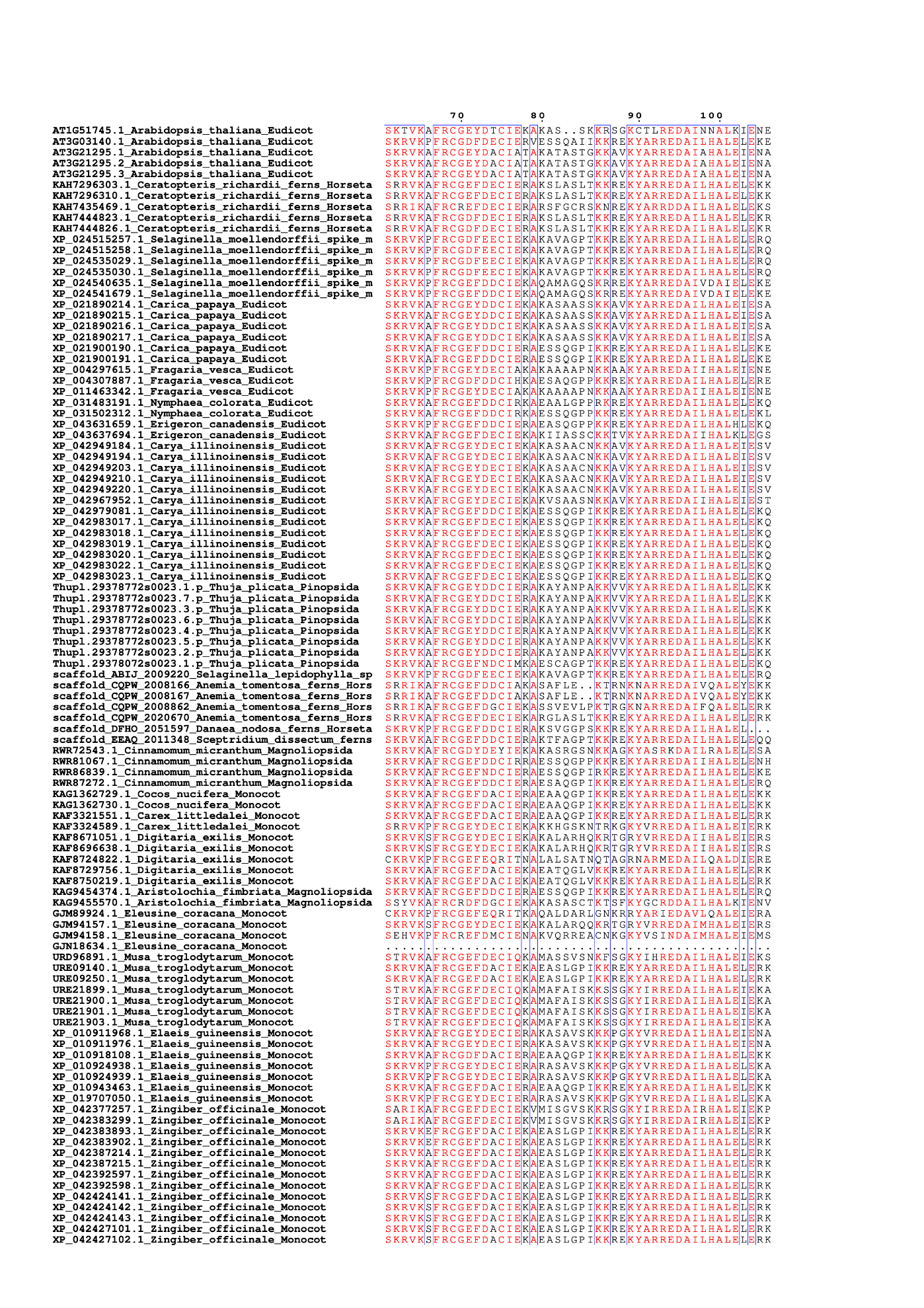


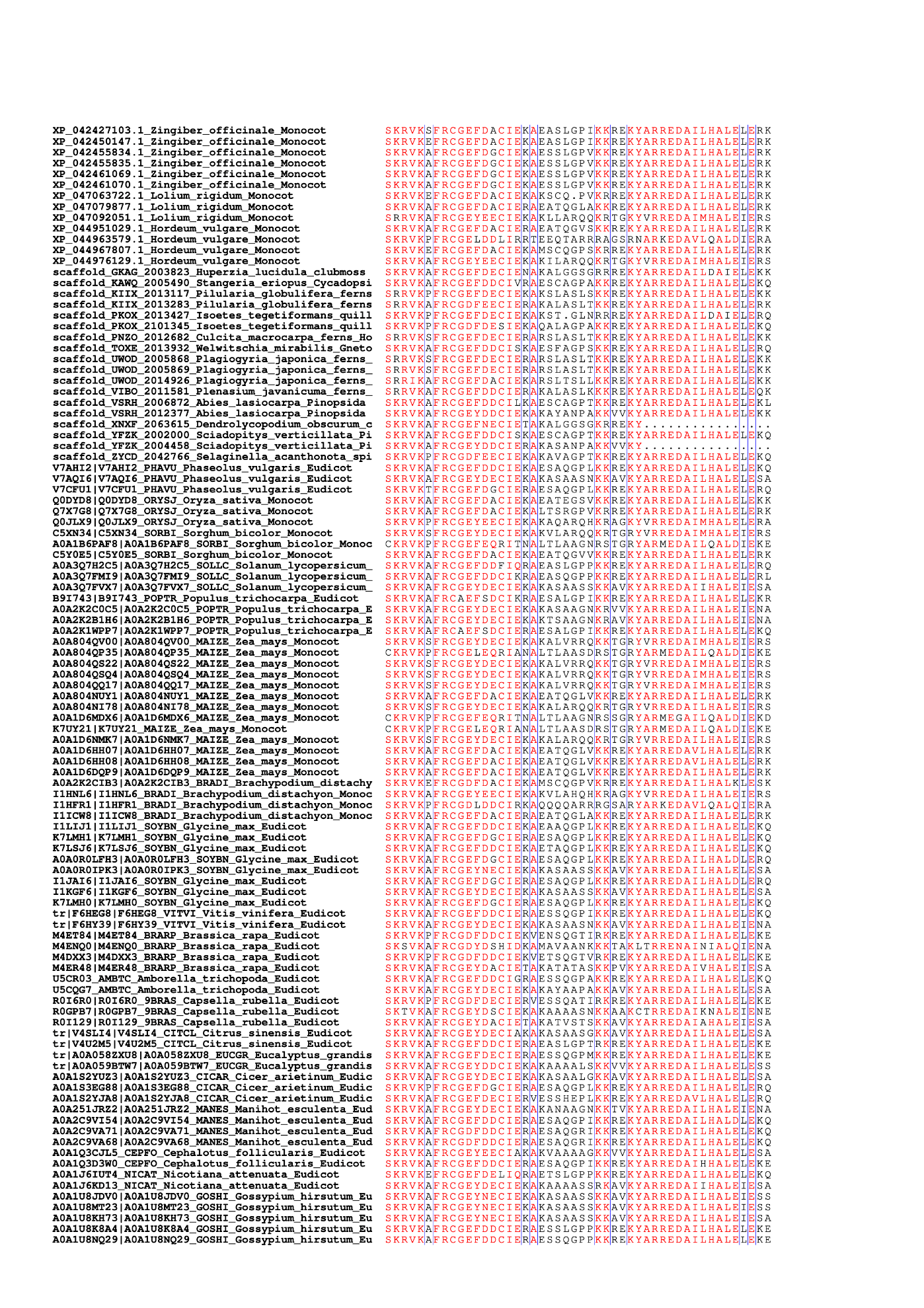


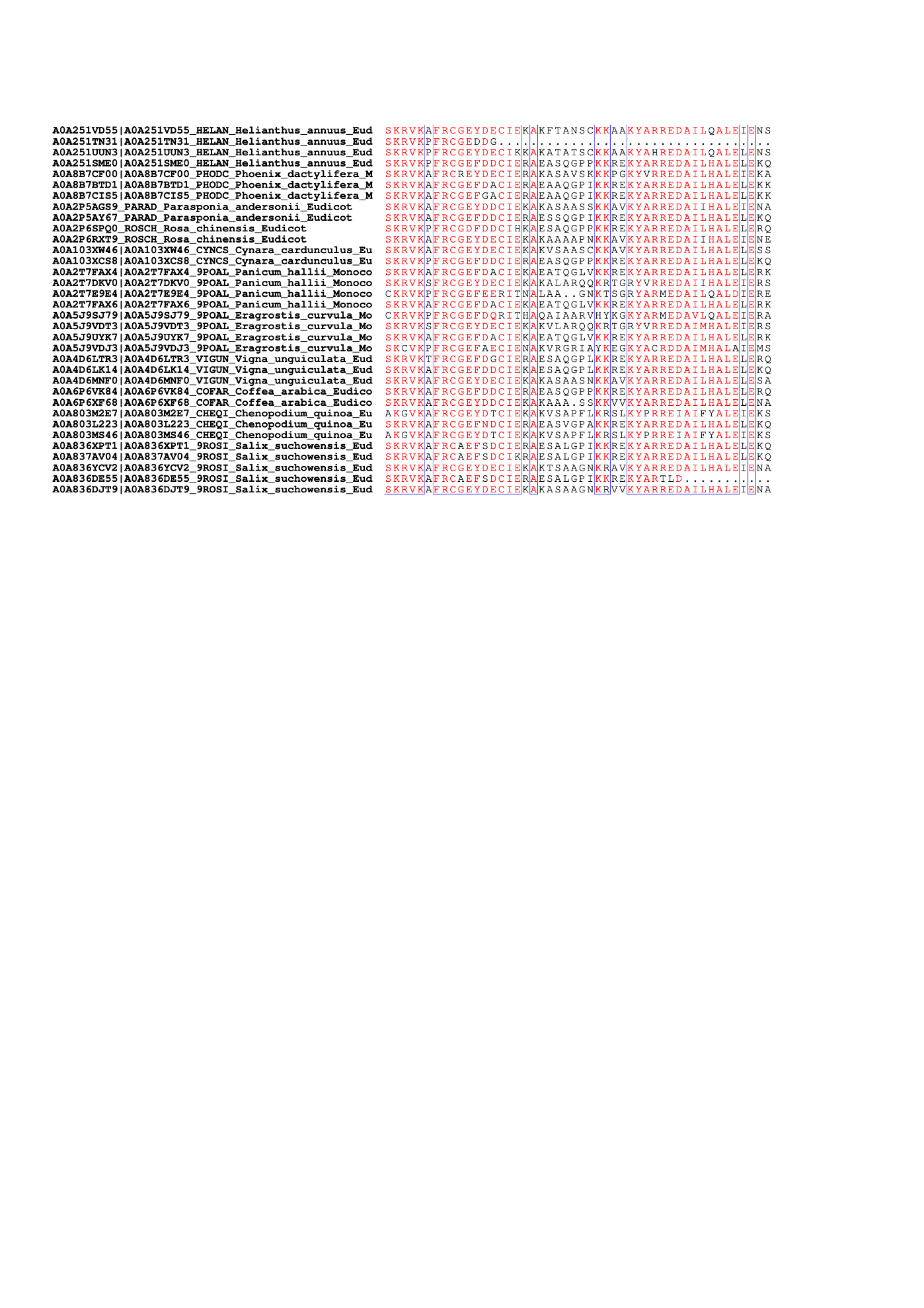


**Supplementary Figure 1.** **Multiple Sequence Alignment (MSA) of the conserved PWWP domain across plant evolution.**

The MSA was generated using MAFFT v7 with default parameters, and ESPript v3 (Robert & Gouet, 2014) was used to highlight conserved regions, secondary structure elements, and other sequence features.


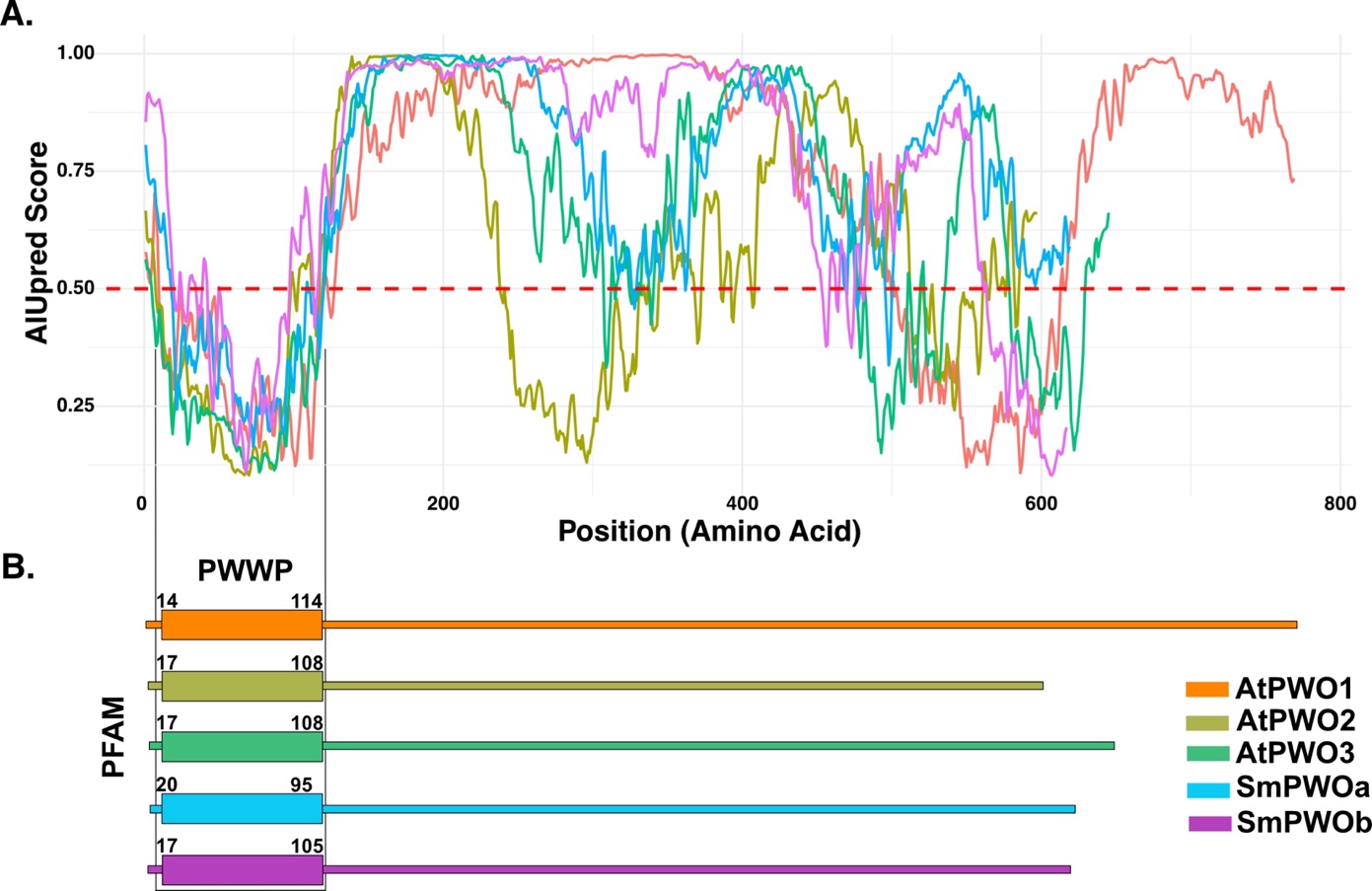


**Supplementary Figure 2. Bioinformatic analysis of Intrinsically Disordered Regions (IDRs) in PWO proteins.**

**A.** Prediction of intrinsically disordered regions in full-length PWO proteins (AtPWO1-3, SmPWOa-b) using Artificial Intelligence-based Unstructured Region Prediction (AIUPred) (Erdős and Dosztányi 2024). Protein regions with an AIUPred score greater than 0.5 are considered disordered. **B.** Position of the PWWP domain as predicted by Pfam (Protein Families Database) (Mistry *et al.*, 2021). The aa positions marking the start and end of the PWWP domain are indicated.


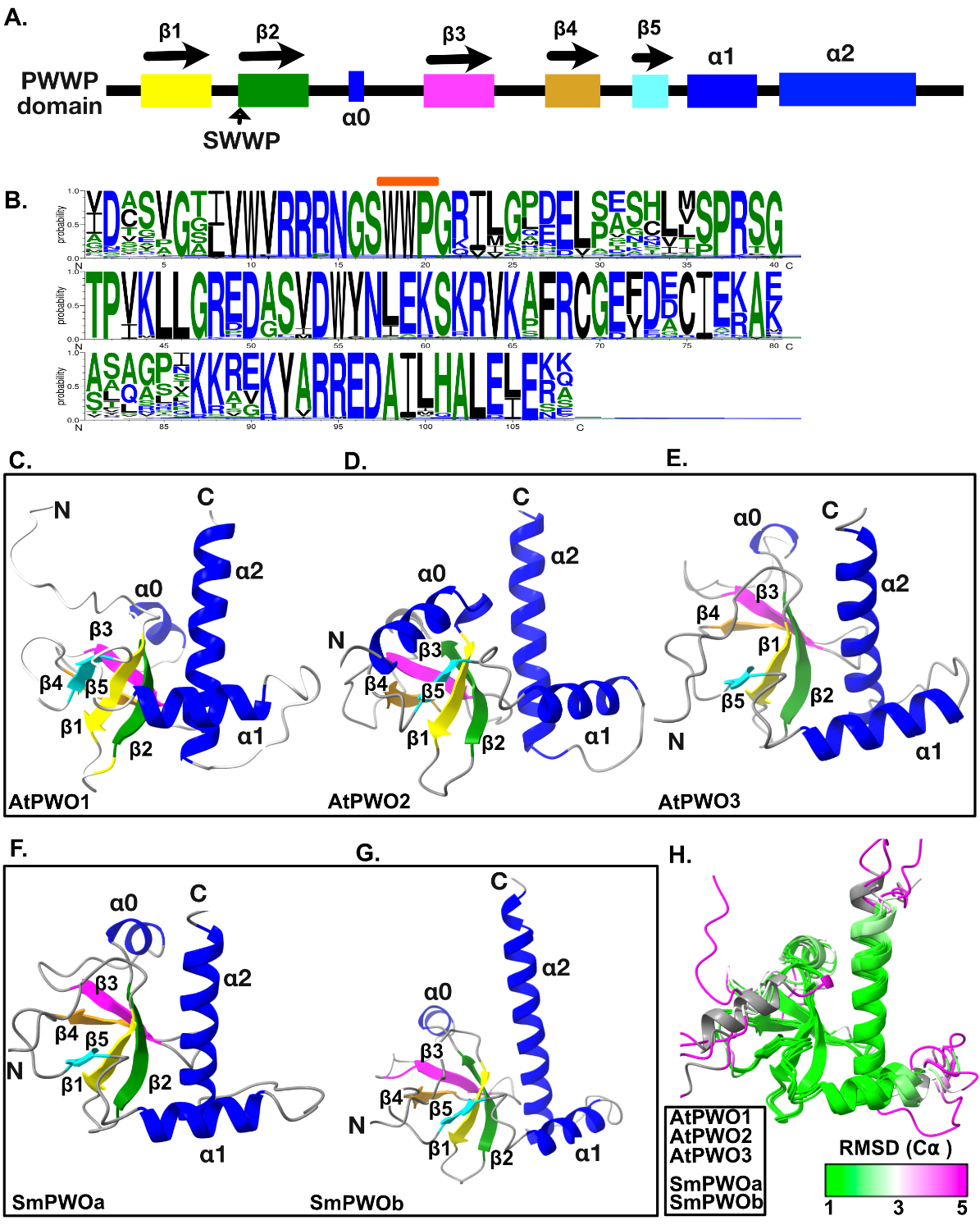


**Supplementary Figure 3. AlphaFold2 structure prediction of *A. thaliana* and *S. moellendorfii* PWO protein PWWP domains.**

**A.** Schematic representation of the PWWP domain, displaying the positioning of β strands (β1: yellow, β2: green, β3: magenta, β4: orange, β5: cyan) and α-helices (α1, α2: blue), with α0 being more variable. The position of SWWP motif at the beginning of the β2 strand is indicated by an arrow. **B**. Consensus sequence plot showing the PWWP domain for all four clades (Clade I-IV), with the orange line indicating the conserved SWWP motif. **C-G.** Structure prediction of the PWWP domain of *A. thaliana* PWO orthologs (AtPWO1, AtPWO2, AtPWO3) (**C-E)** and *S.* *moellendorffii* PWO orthologs (SmPWOa, SmPWOb) **(F-G)**, showing the positioning of secondary structures (β-strands and α-helices) as illustrated in panel A. **H.** Superposition of the PWWP domains of Arabidopsis and S. *moellendorffii* PWO orthologs. The RMSD (Cα) value and color range is displayed, with green indicating values of 1 Å or less, signifying high structural similarity.


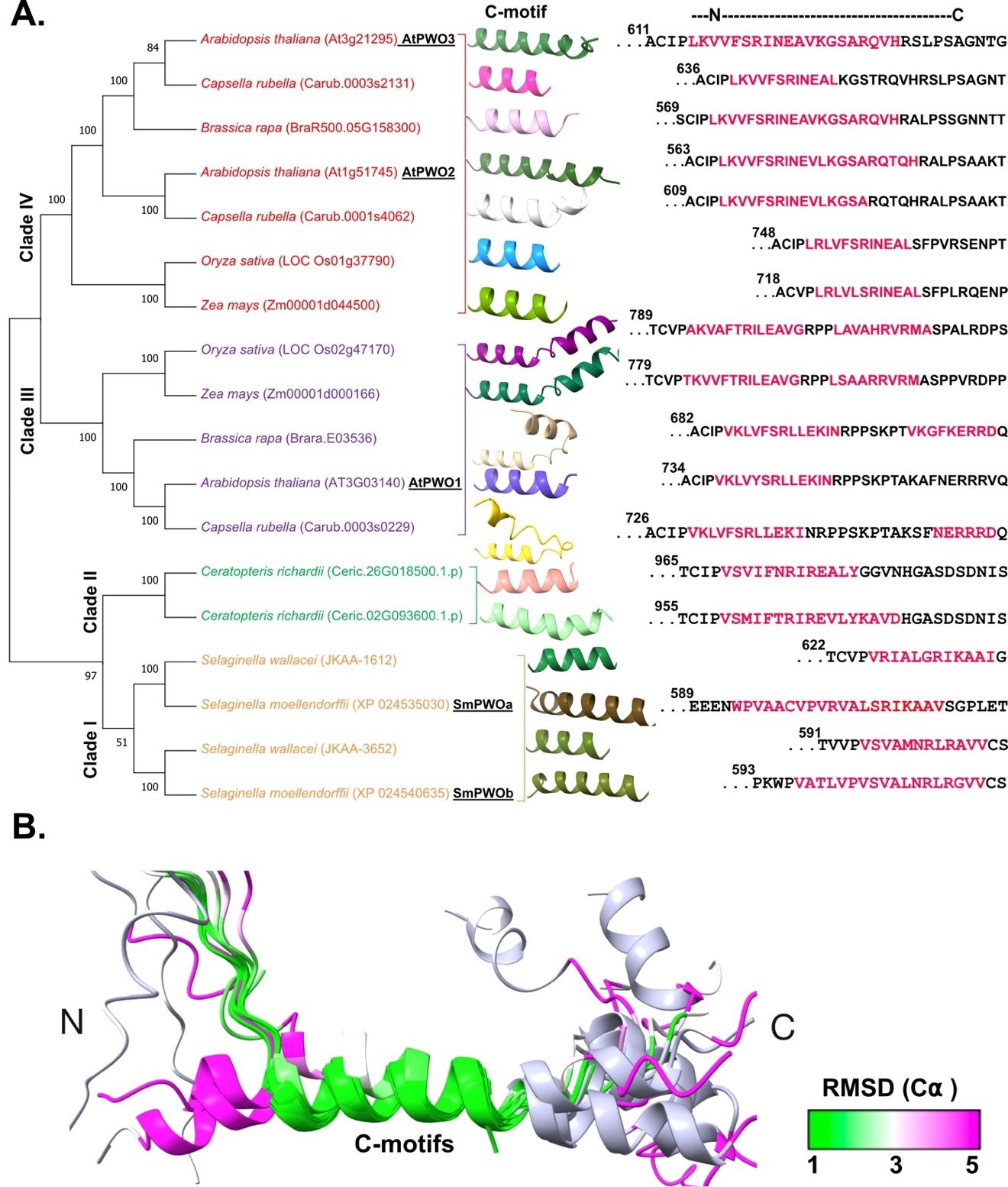


**Supplementary Figure 4. AlphaFold2-based structure prediction of the C-motif in PWO proteins across representative species with PWO clades**.
**A.** The maximum likelihood phylogenetic tree of PWO protein orthologs from 18 selected representative species across the four PWO clades (Clade I, Clade II, Clade III, and Clade IV) depicts the predicted C-motif structures and C-motif sequences, highlighted in pink. **B.** Superposition of all predicted C-motifs in panel 3A is shown, with the RMSD (Cα) color range values displayed. Green represents values of 1 Å or less, indicating high structural similarity.


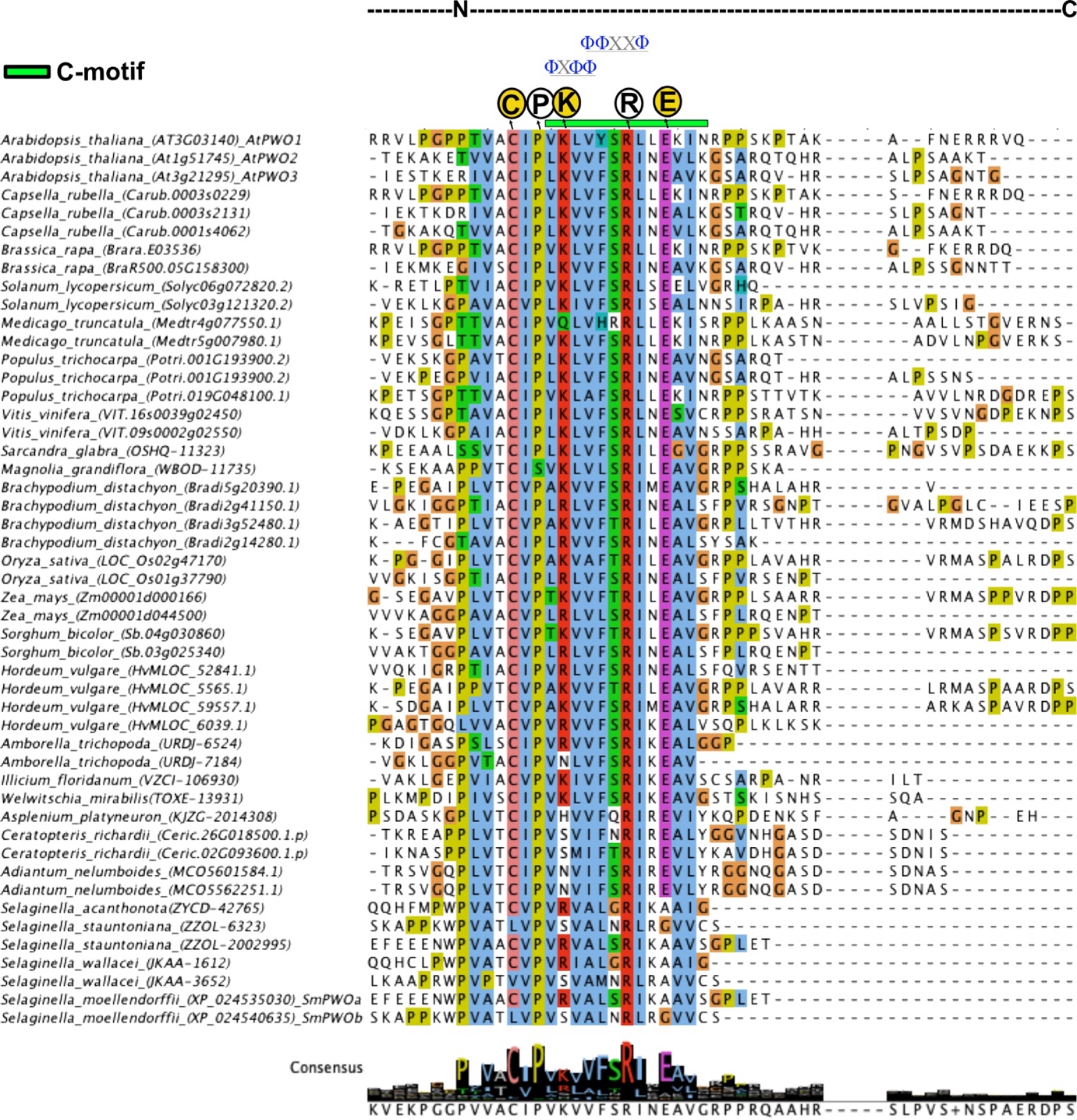


**Supplementary Figure 5.** **Amino acid multiple sequence alignments of PWO C-terminal region including C-motif.**

Sequence alignment was generated using ClustalO, displayed using JalView employing the Clustal color mode. The consensus tracks are calculated by JalView (Waterhouse *et al.*, 2009). The sequence coverage is represented by a black bar. Φ indicates the hydrophobic amino acid (aa) residues. The conserved aa residues are circled black, while a yellow-filled circle indicates lower level of conservation in basal land plants.


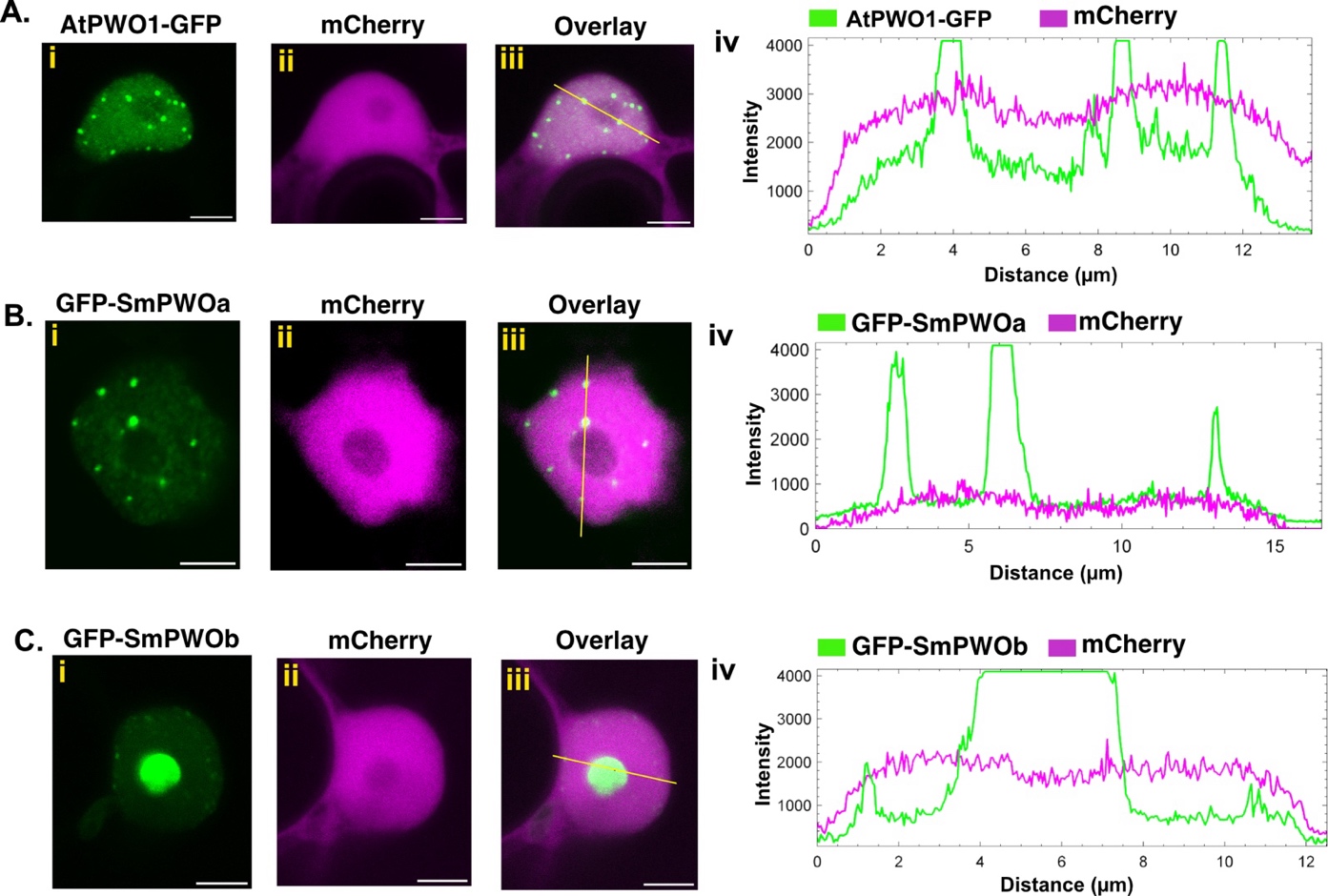


**Supplementary Figure 6. Co-infiltration of PWOs (AtPWO1, SmPWOa, SmPWOb) with empty mCherry**.

Representative confocal microscopy images of nuclei of *N. benthamiana* leaf cells co-infiltrated with *i35S_pro_::mCherry* and **Ai-iii.** *i35S_pro_::AtPWO1-GFP*, **Bi-iii.** *i35S_pro_::GFP-SmPWOa*, and **Ci-iii.** *i35S_pro_::GFP-SmPWOb*. **A-Civ:** Profiles of mCherry and GFP fluorescence intensities along the yellow line in **A-Ciii**. Scale bar = 5 µm.


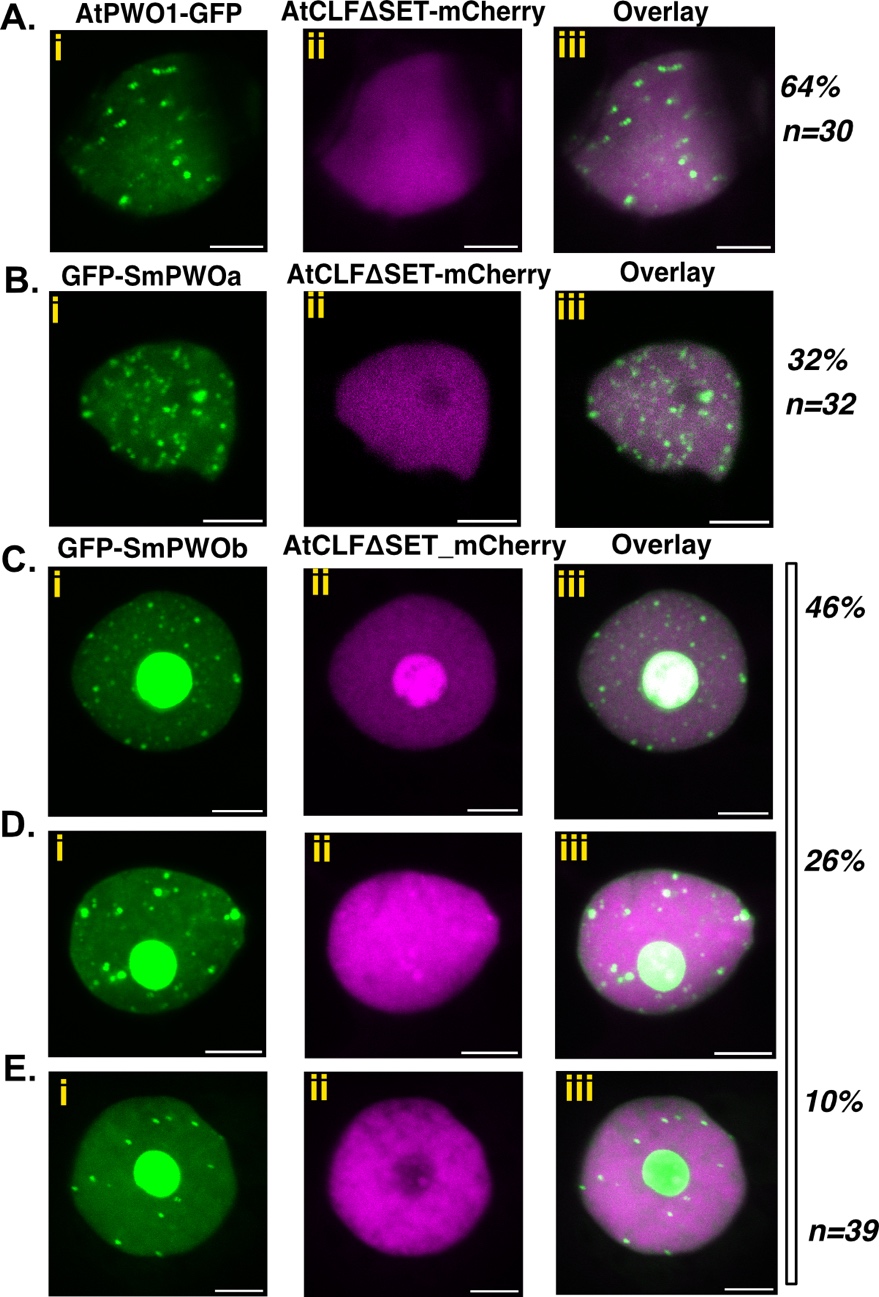


**Supplementary Figure 7. Colocalization of SmPWOs and AtWPO1 with AtCLF in *N. benthamiana*.**

Representative confocal microscopy images showing nuclei of *N. benthamiana* leaf cells infiltrated with *i35S_pro_::mCherry-AtCLFΔSET* and **A.** *i35S_pro_::AtPWO1-GFP*, **B.** *i35S_pro_::GFP-SmPWOa*, **C-D.** *i35S_pro_::GFP-SmPWOb*. The percentage of nuclei with the observed pattern related to the total number of analyzed nuclei (n) is indicated on the right. 'ΔSET' denotes the deletion of the SET domain. Scale bar = 5 µm.


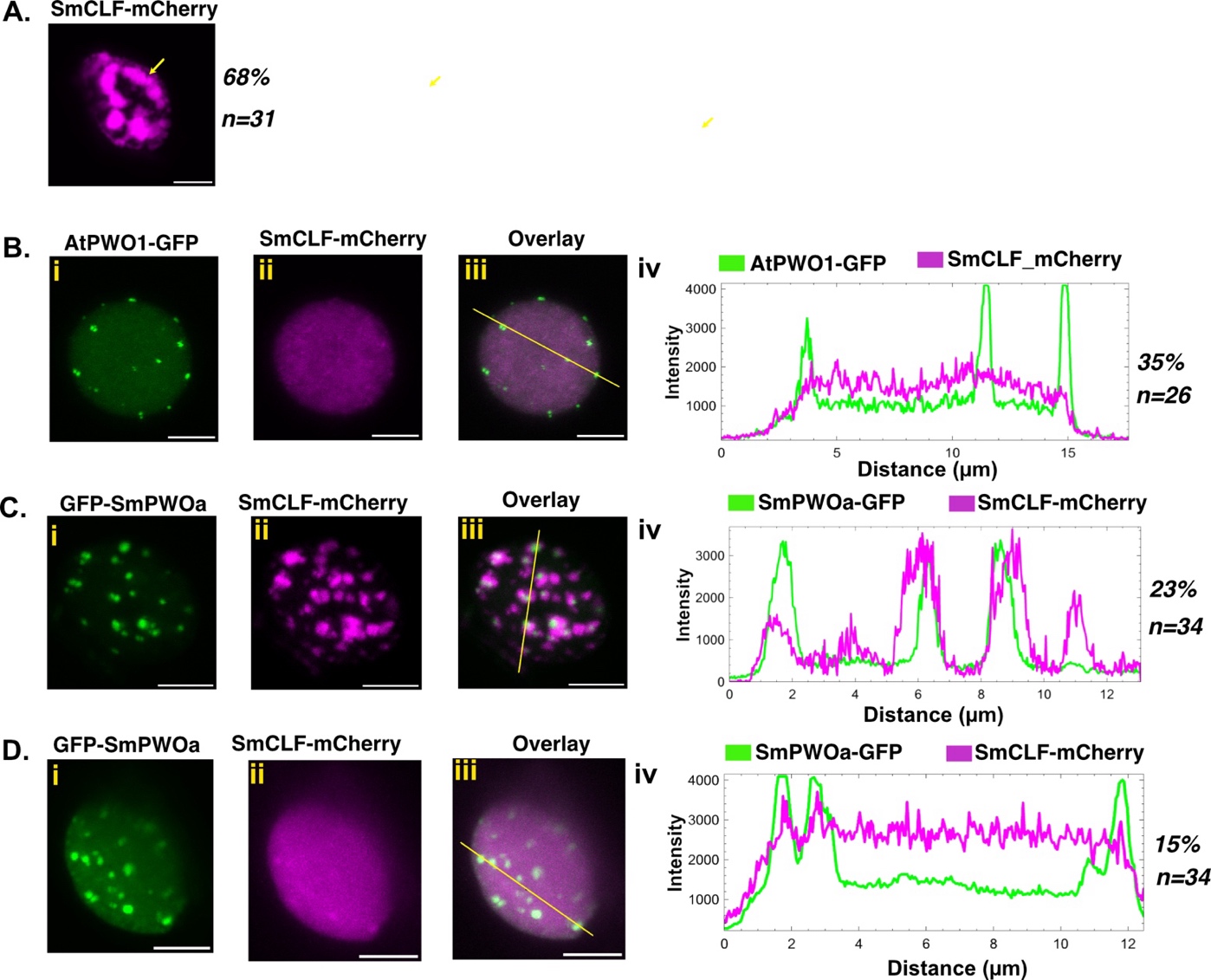


**Supplementary Figure 8. Localization and colocalization of SmCLF with AtPWO1 and SmPWOa in *N. benthamiana***.

Representative confocal microscopy images showing nuclei of *N. benthamiana* leaf cells infiltrated with **A.** *i35S_pro_::SmCLF-GFP*, co-infiltrated with **B.i-iii.** *i35S_pro_::AtPWO1-GFP* and *i35Spro::SmCLF-GFP.* **C-D.i-iii.** *i35S_pro_::GFP-SmPWOa* and *i35S_pro_::SmCLF-GFP*. **B-D.iv:** Profiles of GFP and mCherry fluorescence intensities along the yellow line shown in B-D. iii. The percentage of nuclei with the observed pattern related to the total number of analyzed nuclei (n) is indicated on the right. Arrow indicates larger nuclear patches. Scale bar = 5 µm.


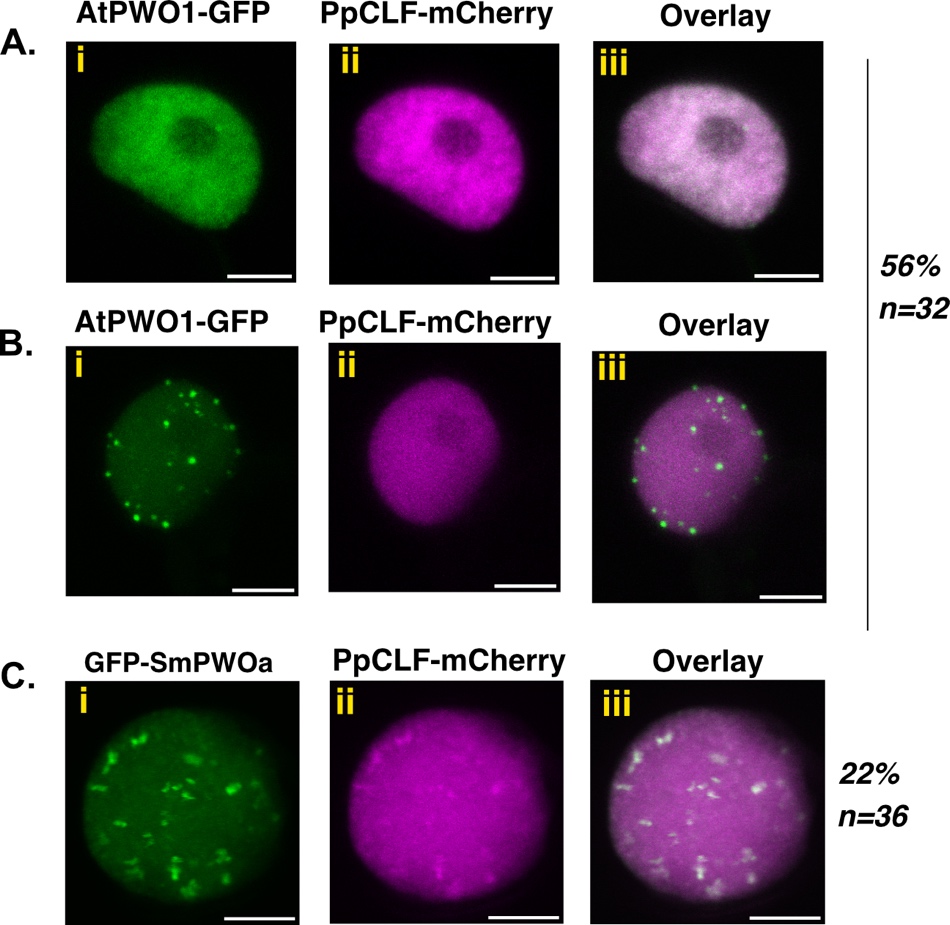


**Supplementary Figure 9. Colocalization of AtPWO1 and SmPWOa with *P. patens* (Pp)CLF in *N. benthamiana*.**

Representative confocal microscopy images showing nuclei of *N. benthamiana* leaf cells co-infiltrated with **A-B.i-iii.** *i35S_pro_::AtPWO1-GFP* and *i35S_pro_::PpCLF-mCherry*. **C.i-iii.** *i35S_pro_::GFP-SmPWOa* and *i35S_pro_::PpCLF-mCherry*. The percentage of nuclei with the observed pattern related to the total number of analyzed nuclei (n) is indicated on the right. Scale bar = 5 µm.


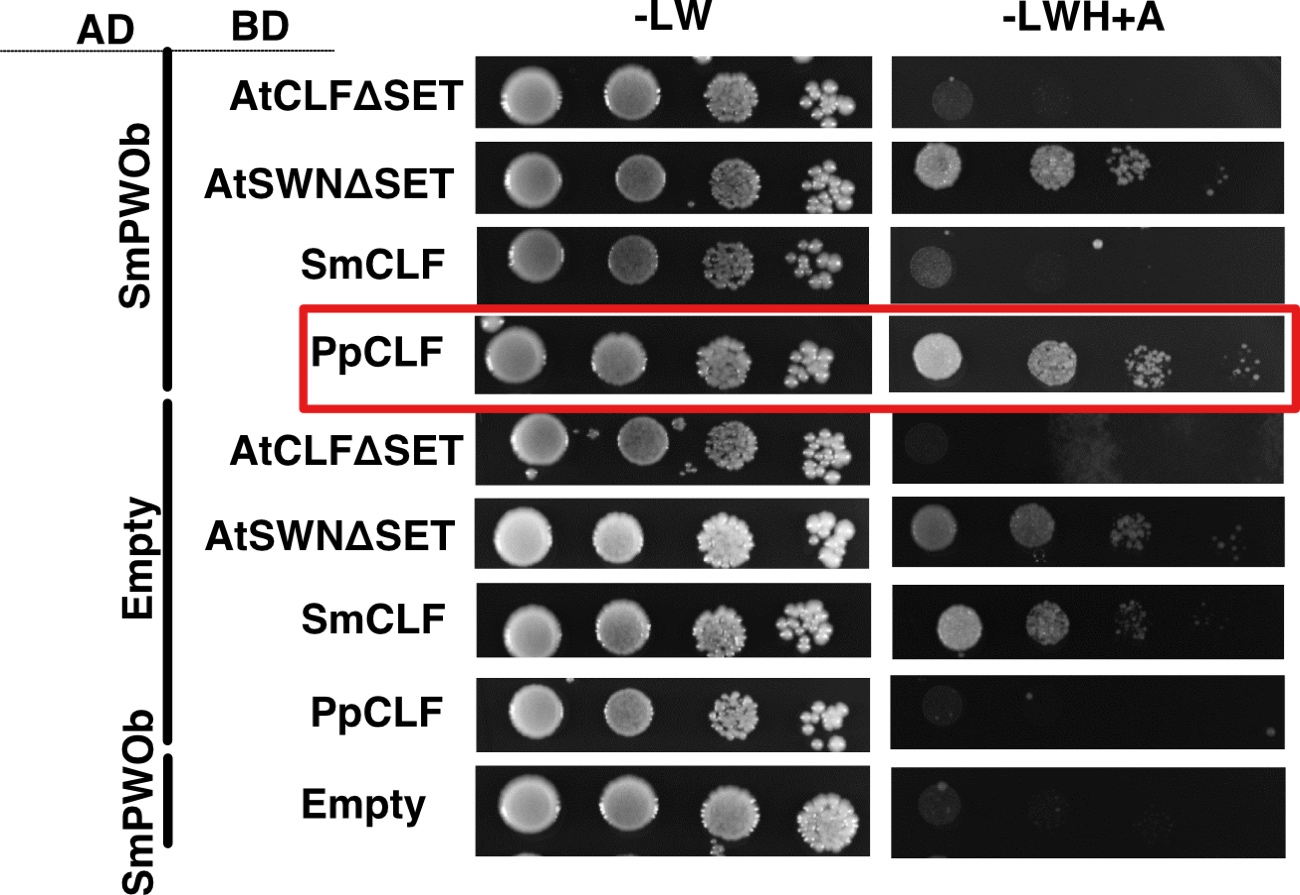


**Supplementary Figure 10. Yeast two-hybrid assays for SmPWOb and PRC2 catalytic subunit interactions.**

Interactions between SmPWOb and AtCLF, AtSWN, PpCLF, and SmCLF were tested on low-stringency medium lacking leucine, tryptophan, histidine, and containing adenine (−LWH + A). The red box highlights a weak interaction between SmPWOb and PpCLF. Interactions were only assessed as positive if no autoactivation was observed (combination of AD “Empty” and BD CLF/SWN or AD SmPWOb and BD “Empty”).


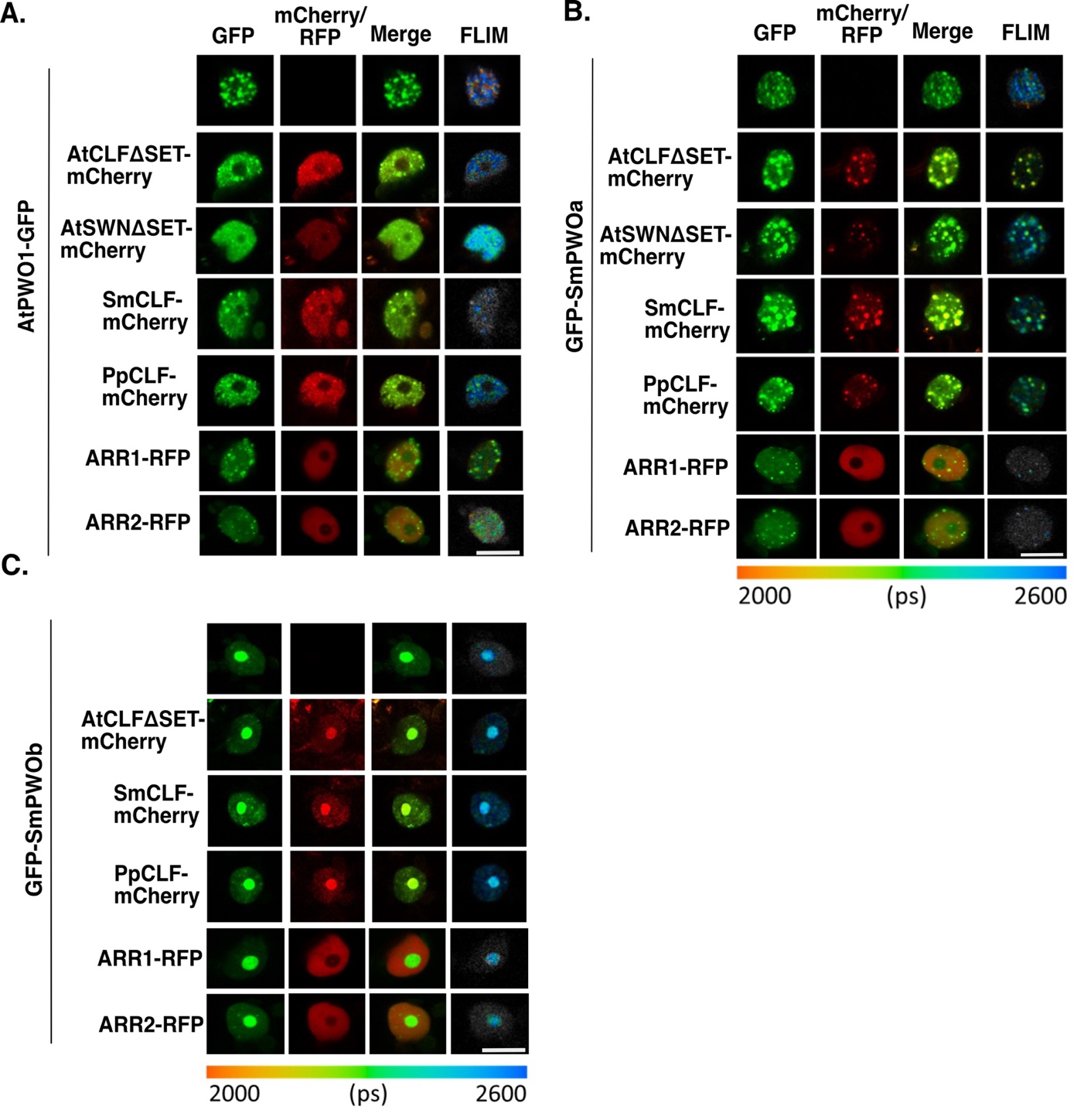


**Supplementary Figure 11. FLIM-FRET confocal microscopy images for PWOs and CLF orthologs.**

**A. AtPWO1, B. SmPWOa, and C. SmPWOb interactions with the PRC2 catalytic subunits from Arabidopsis (AtCLF, AtSWN), *S. moellendorffii* (SmCLF), and *P. patens* (PpCLF).**Scale bar = 10 μm. The FLIM-FRET data are displayed using a lifetime Look-Up Table (LUT). ARR1/2-RFP serves as negative controls.


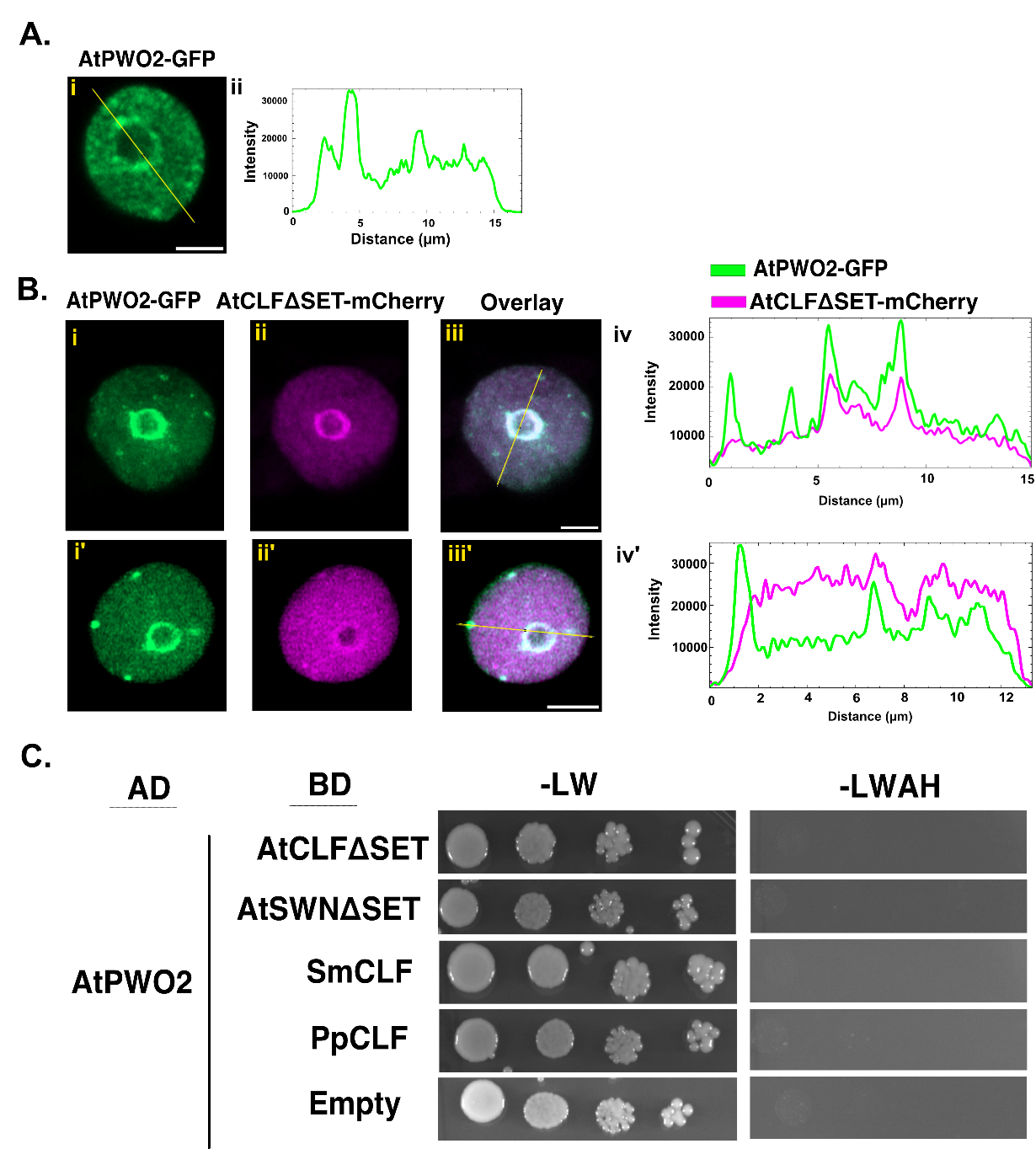


**Supplementary Figure 12. PWO2 localization, colocalization, and interaction with CLF**.

**A-B.** Representative confocal microscopy images showing nuclei of *N. benthamiana* leaf cells infiltrated with **Ai-ii**. *i35Spro::AtPWO2-GFP* and **Bi-iv’.** Co-infiltration of *i35Spro::AtPWO2-GFP* with *i35Spro::PpCLF-mCherry*. Profiles of mCherry and GFP fluorescence intensities along the yellow line are shown in **A-ii, Biv and Biv’.** Scale bar = 5 µm. **C.** Y2H interaction analyses of AtPWO2 with AtCLFΔSET, AtSWNΔSET, SmCLF and PpCLF. The interactions were evaluated by growing transformed yeast cells on non-selective medium (-LW; lacking leucine and tryptophan) to facilitate plasmid co-transformation and on selective medium (-LWAH; lacking leucine, tryptophan, adenine, and histidine) to activate the reporter gene. BD, GAL4 DNA-binding domain; AD, GAL4 DNA-activation domain fusion. Constructs containing CLF/SWN with SET domain deletions are denoted as ΔSET.


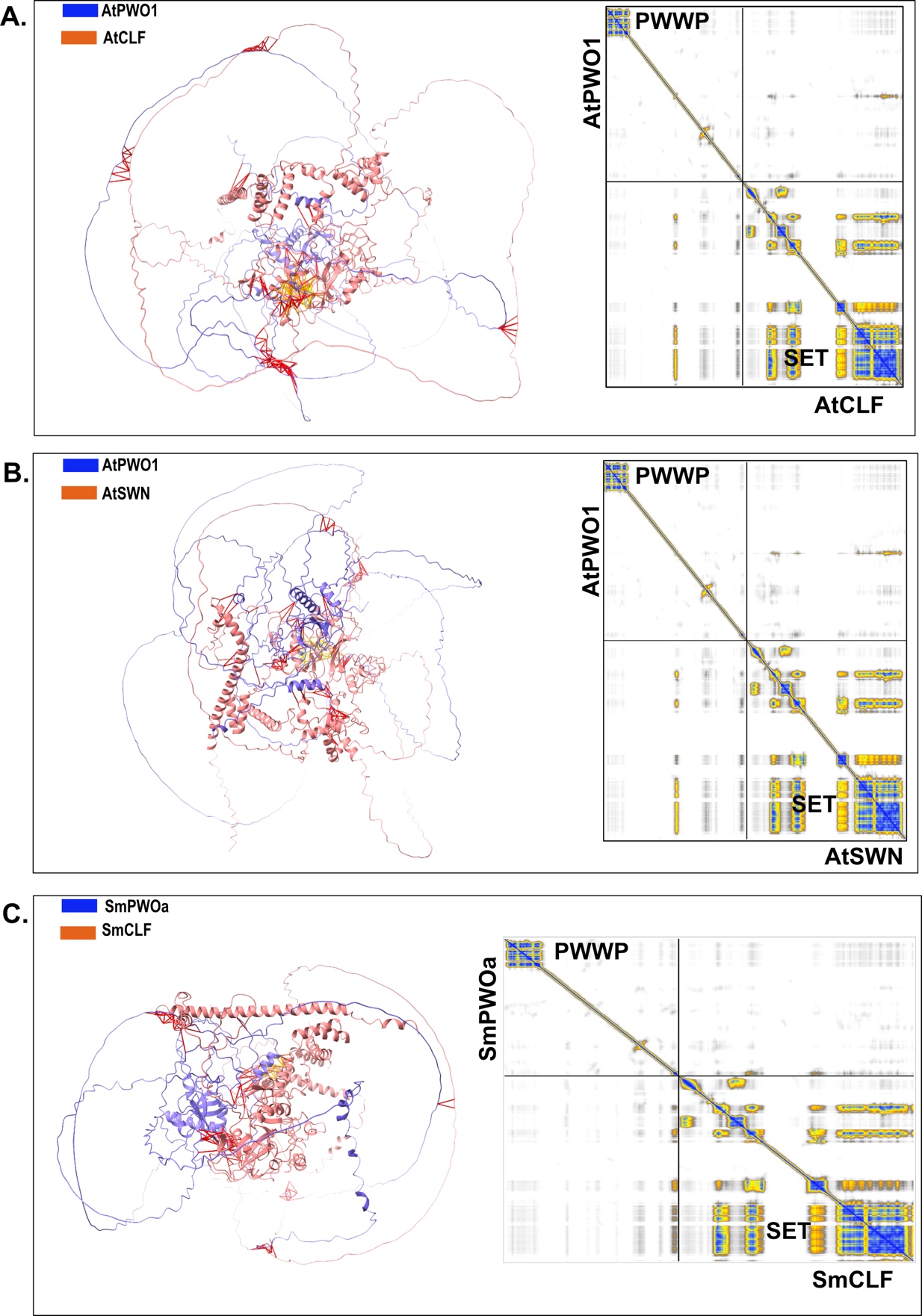


**Supplementary Figure 13. AlphaFold2-Multimer (AF2-M)-based prediction of interaction surfaces between PWOs and CLF or SWN in *A. thaliana* and *S. moellendorffii.***

AF2-M prediction of the interaction surface between full-length **A.** AtPWO1 and AtCLF, **B.** AtPWO1 and AtSWN, **C.** SmPWOa and SmCLF, along with the Predicted Aligned Error (PEA) plot for rank 1, for each of the combinations.


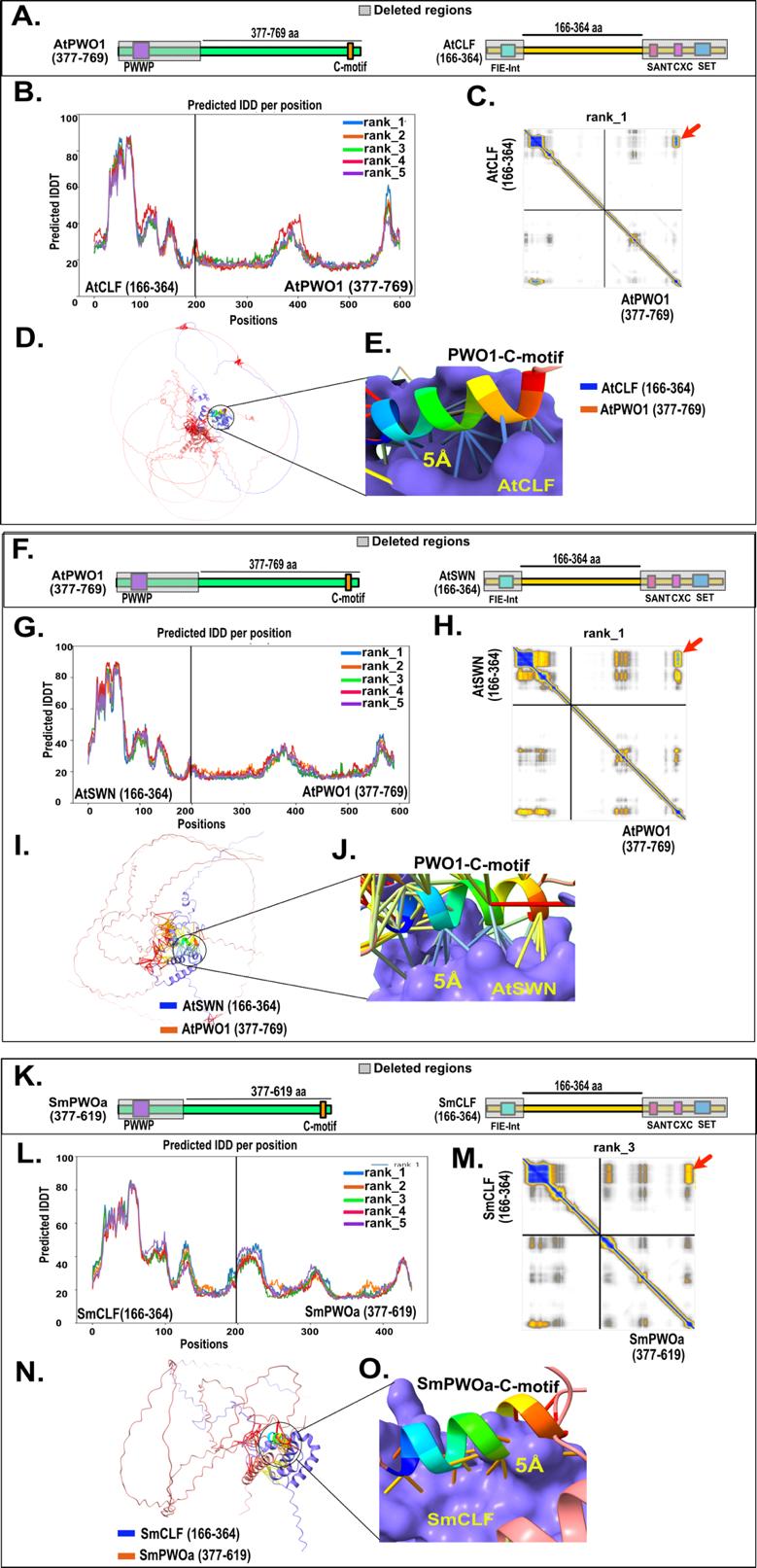


**Supplementary Figure 14. AF2-M-based prediction of the interaction surface between truncated PWOs and CLF/SWN.**

**A.** Schematic diagram showing the truncated fragments of AtPWO1 and AtCLF used for interaction prediction. **B.** pLDTT graph showing values for five models for AtPWO1 (377-769 aa) interaction with AtCLF (166-364 aa). **C.** PAE plot displaying the rank 1 models from panel B with noticeable local interactions in PAE maps, outlined by red arrow. **D.** 3D view of the predicted AtPWO1–AtCLF interaction from panel B rank 1 with, **E.** Zoom-in view of the interaction interface, highlighting the interaction between the AtPWO1 C-motif (rainbow-colored) and AtCLF (blue surface). **F.** Schematic diagram showing the truncated fragments of AtPWO1 and AtSWN used for interaction prediction**.** **G.** pLDTT graph showing values for five models for AtPWO1 (377-769 aa) interaction with AtSWN (166-364 aa). **H.** PEA plot displaying the rank 1 models from panel G with local interactions in PAE maps, outlined by red arrow. **I.** 3D view of the predicted AtPWO1–AtSWN complex shown from panel G rank 1 with, **J.** Zoom-in view of the interaction interface, highlighting the interaction between the AtPWO1 C-tail (rainbow-colored) and AtSWN (blue surface). **K.** Schematic diagram showing the truncated fragments of SmPWOa and SmCLF used for interaction prediction**.** **L.** pLDTT graph showing values for five models for SmPWOa (377-619 aa) interaction with SmCLF (166-364 aa). **M.** PEA plot displaying the rank 3 models from panel L with local interactions in PAE maps, outlined by red arrow. **N.** 3D view of the predicted SmPWOa–SmCLF complex shown in panel L rank 3 with, **O.** Zoom-in view of the interaction interface, highlighting the interaction between the SmPWOa C-tail (rainbow-colored) and SmCLF (blue surface).


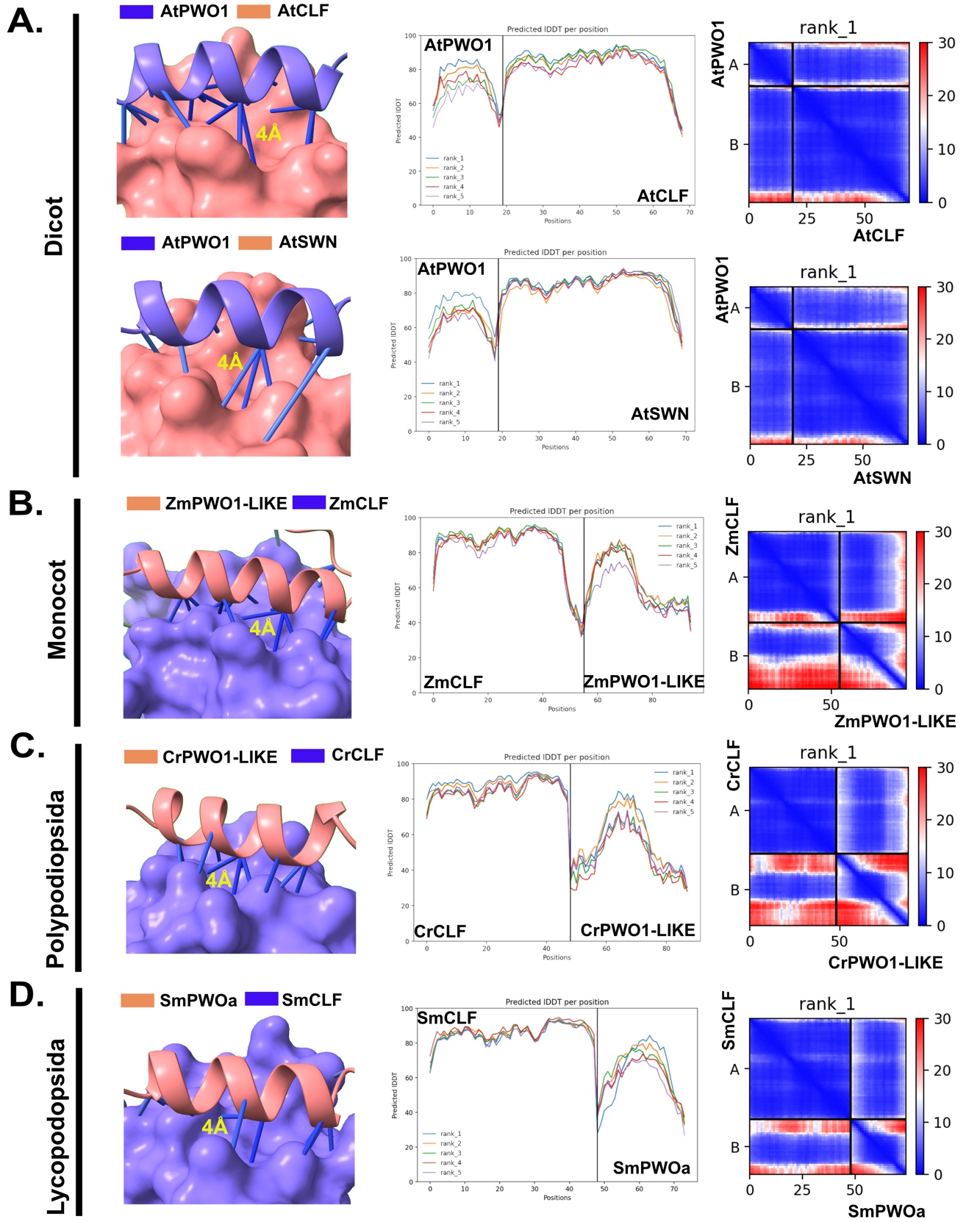


**Supplementary Figure 15. AF2-M prediction of interaction surfaces between PWOs and PRC2 catalytic subunit (CLF) across plant species.**

Predicted interaction surfaces between PWOs and the PRC2 catalytic subunit (CLF) in selected representative plant species. AF2-M-based predictions of specific interaction interfaces are shown for: **A.** AtPWO1 (735–754 aa) with AtCLF (198–248 aa) and AtPWO1 (735–754 aa) with AtSWN (185–236 aa). **B.** ZmPWO1 (777–817 aa) with ZmCLF (223–281 aa). **C.** CrPWO1 (756–796 aa) with CrCLF (221–269 aa). **D.** SmPWOa (593–691 aa) with SmCLF (182–230 aa). Each panel includes a pLDDT graph showing the confidence values across five models and a PAE plot displaying the top-ranked model for each interaction.


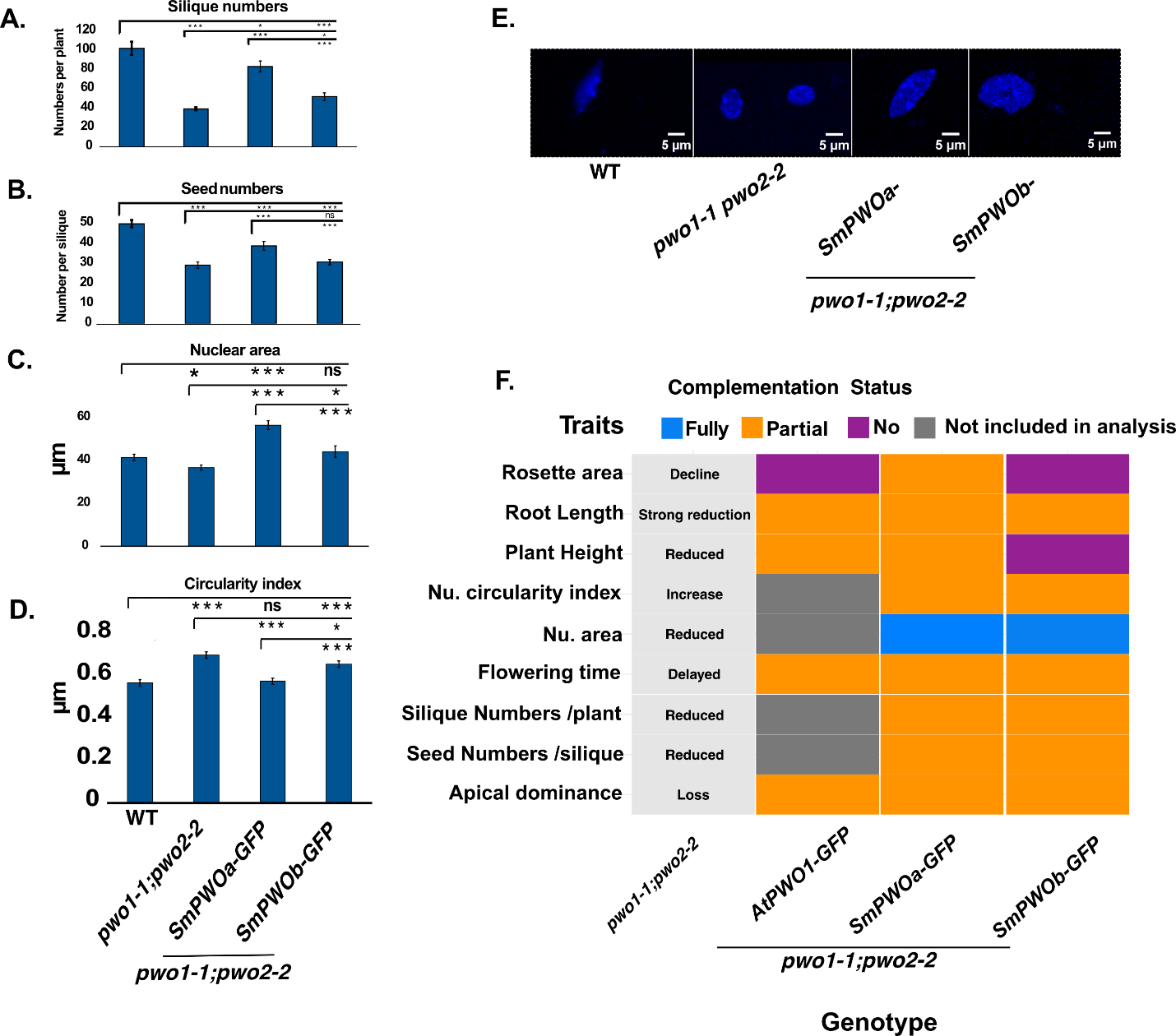


**Supplementary Figure 16. Role of PWOs in Arabidopsis development and nuclear morphology.**

**A.** Number of siliques per plant (n = 10); **B.** Seed number per silique (n = 10); **C - D.** Nuclear morphology (n = 100) of the plant lines Col-0, *pwo1-1;pwo2-2*, *2x35S_pro_::SmPWOa-GFP*/*pwo1-1; pwo2-2*, and *2x35S_pro_::SmPWOb-GFP*/*pwo1-1;pwo2-2* lines. The nuclear area **C.** and circularity **D.** measurements show the largest area of a particular nucleus in the z-stack (n = 100). **E.** Representative DAPI-stained nuclei from the analysed genotypes. Error bars correspond to ±SD. Asterisks represent p-values: ***p ≤ 0.001, **p ≤ 0.01, *p ≤ 0.05. The two-tailed Student’s t-test was applied to calculate the significance level. Scale bars = 5 µm. **F.** Summary of all developmental defects (Figure 9 and Supplementary Figure 15) observed in *pwo1-1;pwo2-2* mutants and their complementation status (partial, fully, or none) by the *2x35S_pro_::AtPWO1-GFP 2x35S_pro_::SmPWOa-GFP*/*pwo1-1; pwo2-2*, and *2x35S_pro_::SmPWOb-GFP*/*pwo1-1;pwo2-2*, transgenic lines.


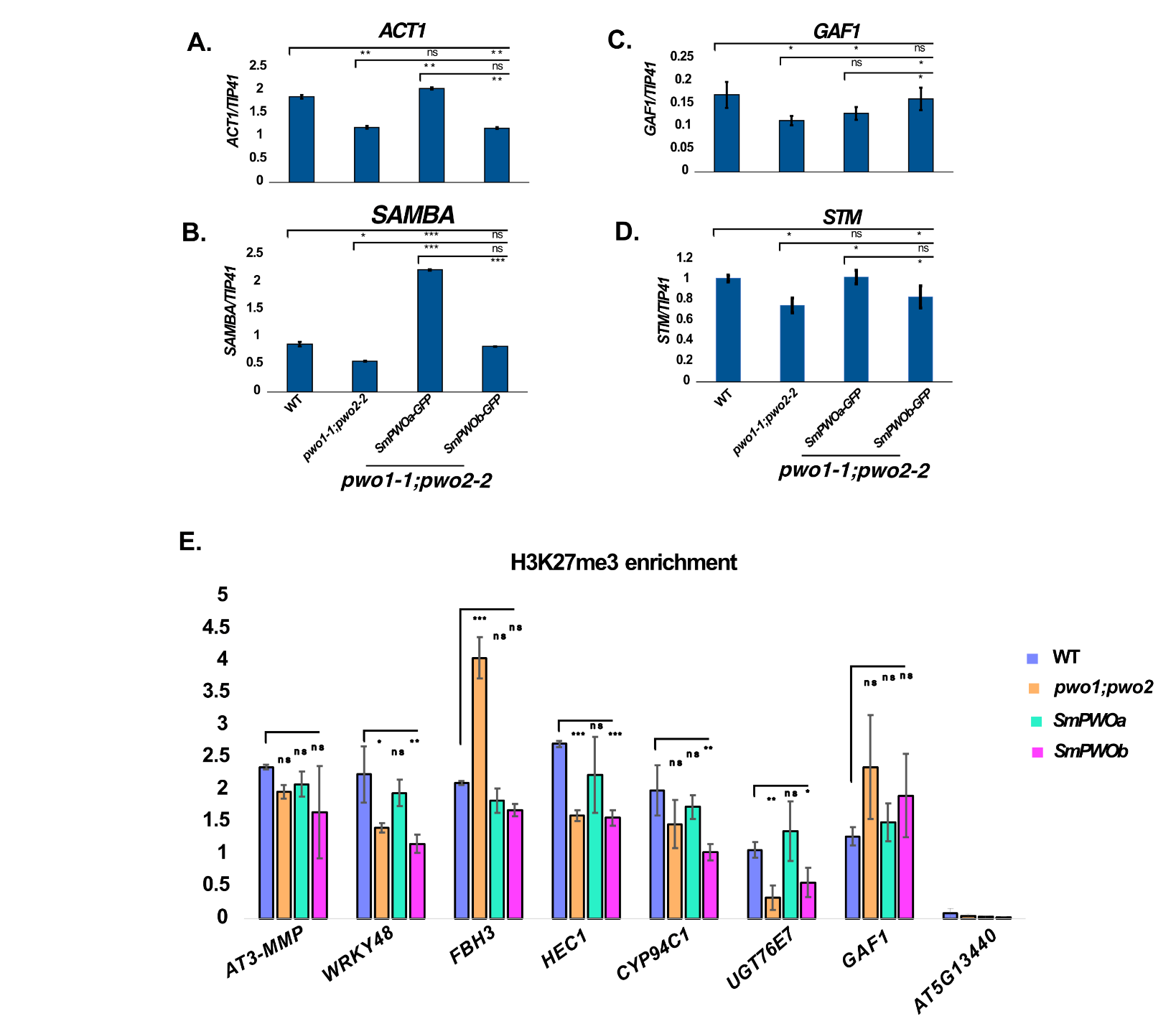


**Supplementary Figure 17. RT-qPCR analyses of genes mis-regulated in *pwo1-1;pwo2-2* and levels of H3K27me3 at PWO1 targets in *pwo1;pwo2* and complemented lines.**
Quantitative RT-qPCR analyses of **A**. *ACTINE 1* (*ACT1*), **B**. *SAMBA*, **C**. *GAMETOPHYTE DEFECTIVE 1* (*GAF1*) and **D**. *SHOOT MERISTEMLESS* (*STM*) expression levels in the genotypes Col-0, *pwo1-1;pwo2-2*, *2x35S_pro_::SmPWOa-GFP*/*pwo1-1;pwo2-2*, and *2x35S_pro_::SmPWOb-GFP*/*pwo1-1;pwo2-2* lines. Results were normalized using *TAP42 INTERACTING PROTEIN OF 41 KDA* (*TIP41*) as housekeeping gene. All samples were from 10-day-old seedlings. Data represent the mean values of three replicates. Two biological repeats were conducted. **E.** H3K27me3 enrichment at *METALLOENDOPROTEINASE 3-MMP* (*AT3-MMP*)*, WRKY48, FLOWERING BHLH 3* (*FBH3*), *HECATE1* (*HEC1*)*, CYP94C1, UDP-GLYCOSYLTRANSFERASE 76E7* (*UGT76E7*)*,* and *GAMETOPHYTE DEFECTIVE 1* (*GAF1*) in Col-0, *pwo1-1;pwo2-2* (*pwo1;pwo2*), *2x35S_pro_::SmPWOa-GFP*/*pwo1-1;pwo2-2* (*SmPWOa*), and *2x35S_pro_::SmPWOb-GFP*/*pwo1-1;pwo2-2* (*SmPWOb*)  lines. AT5G13440 was selected as a ChIP negative control locus, not occupied by H3K27me3. At least two biological replicates and three technical repeats were used. Error bars correspond to ±SD. Asterisks represent p-values: ***p ≤ 0.001, **p ≤ 0.01, *p ≤ 0.05. The two-tailed Student’s t-test was applied to calculate the significance level.

**References**

**Erdős G, Dosztányi Z**. **2024**. AIUPred: combining energy estimation with deep learning for the enhanced prediction of protein disorder. *Nucleic Acids Research* **52**: W176–W181.

**Mistry J, Chuguransky S, Williams L, Qureshi M, Salazar GA, Sonnhammer ELL, Tosatto SCE, Paladin L, Raj S, Richardson LJ, *et al.*** **2021**. Pfam: The protein families database in 2021. *Nucleic Acids Research* **49**: D412–D419.

**Robert X, Gouet P**. **2014**. Deciphering key features in protein structures with the new ENDscript server. *Nucleic Acids Research* **42**: W320–W324.

**Waterhouse AM, Procter JB, Martin DMA, Clamp M, Barton GJ**. **2009**. Jalview Version 2—a multiple sequence alignment editor and analysis workbench. *Bioinformatics* **25**: 1189–1191.
